# Anther development in *Arabidopsis thaliana* involves symplastic isolation and apoplastic gating of the tapetum-middle layer interface

**DOI:** 10.1242/dev.200596

**Published:** 2022-11-16

**Authors:** Jekaterina Truskina, Sophy Boeuf, Joan Renard, Tonni Grube Andersen, Niko Geldner, Gwyneth Ingram

**Affiliations:** ^1^ Laboratoire Reproduction et Développement des Plantes, ENS de Lyon, CNRS, INRAE, UCBL, F-69342 Lyon, France; ^2^ Department of Cell and Metabolic Biology, Leibniz Institute of Plant Biochemistry, D-06120 Halle (Saale), Germany; ^3^ Instituto de Biología Molecular y Celular de Plantas, Universitat Politècnica de València-Consejo Superior de Investigaciones Científicas, Camino de Vera, Valencia 46022, Spain; ^4^ Department for Plant-microbe Interactions, Max Planck Institute for Plant Breeding Research, 50829 Cologne, Germany; ^5^ Department of Plant Molecular Biology, University of Lausanne, 1015 Lausanne, Switzerland

**Keywords:** Anther, Apoplastic barrier, Lignin, Tapetum, *Arabidopsis*

## Abstract

During flowering plant reproduction, anthers produce pollen grains, the development of which is supported by the tapetum, a nourishing maternal tissue that also contributes non-cell-autonomously to the pollen wall, the resistant external layer on the pollen surface. How the anther restricts movement of the tapetum-derived pollen wall components, while allowing metabolites such as sugars and amino acids to reach the developing pollen, remains unknown. Here, we show experimentally that in *arabidopsis thaliana* the tapetum and developing pollen are symplastically isolated from each other, and from other sporophytic tissues, from meiosis onwards. We show that the peritapetal strip, an apoplastic structure, separates the tapetum and the pollen grains from other anther cell layers and can prevent the apoplastic diffusion of fluorescent proteins, again from meiosis onwards. The formation and selective barrier functions of the peritapetal strip require two NADPH oxidases, RBOHE and RBOHC, which play a key role in pollen formation. Our results suggest that, together with symplastic isolation, gating of the apoplast around the tapetum may help generate metabolically distinct anther compartments.

## Teaser

**Summary:** Apoplastic movement between the tapetum and middle layer of the developing *Arabidopsis* anther is gated by a previously uncharacterised apoplastic barrier, the peritapetal strip.

## INTRODUCTION

In angiosperms, male gametophytes, called pollen grains, are produced inside specialised floral organs, the anthers. In the model plant *Arabidopsis*, as in most angiosperms, during pollen formation diploid precursor cells undergo meiosis to produce small haploid cells (microspores) that are initially held together as tetrads by callose, which is subsequently degraded, releasing the microspores into the gel-like locular matrix, the composition of which is unclear. Microspores subsequently undergo extensive growth and maturation and gradually acquire the tough external pollen wall that will enable the mature pollen grains to survive the effects of desiccation, solar radiation and other environmental stresses. The locular matrix is surrounded by a layer of highly metabolically active maternal cells, the tapetum, which, after producing key enzymes required for microspore release (Bucciaglia and Smith, 1994; Hird et al., 1993; Stieglitz and Stern, 1973), supplies the pollen with most of the materials necessary for its development, including the components of the protective pollen wall (sporopollenin precursors) (Quilichini et al., 2015). Just before pollen maturation is completed, the tapetum undergoes programmed cell death, releasing a myriad of substances into the locular matrix, many of which associate with the sporopollenin scaffold at the pollen grain surface to complete the pollen wall (Gómez et al., 2015). Thus, the tapetum and future pollen grains can be functionally described as the zone of active pollen development (ZPD).

**Fig. 1.**
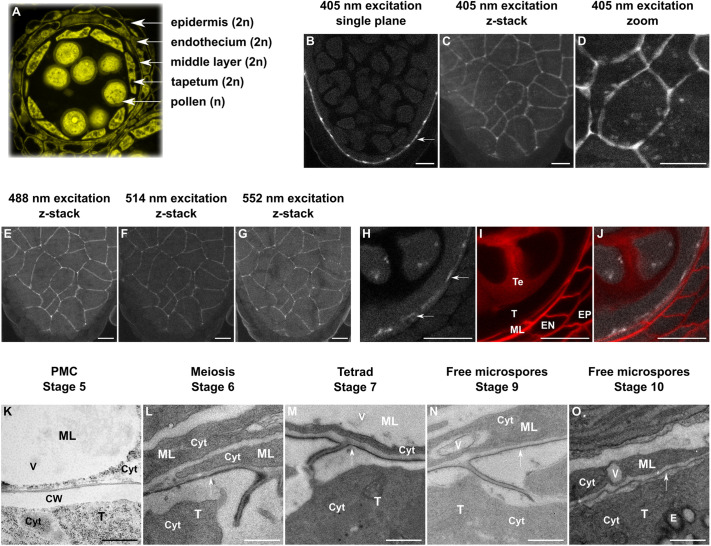
**Visualisation of the PTS in *Arabidopsis thaliana* anthers.** (A) In *Arabidopsis thaliana*, the developing pollen is surrounded by the four diploid sporophytic cell layers: tapetum, middle layer, endothecium and epidermis. (B-G) The peritapetal strip in anthers at the tetrad stage (post-meiosis) excited at 405 nm (B-D), 488 nm (E), 514 nm (F) and 552 nm (G). (H-J) Localisation of the PTS in the anthers (J); the PTS was visualised using 514 nm laser (H) and the anther cell walls were stained with the SCRI Renaissance Stain 2200 (I). (K-O) TEM of the anther showing the middle layer–tapetum interface at the indicated stages of anther development (staging according to Sanders et al., 1999). Arrows indicate the PTS. CW, cell wall; Cyt, cytoplasm; E, elaioplast; EN, endothecium; EP, epidermis; ML, middle layer; PMC, pollen mother cell stage of pollen development; T, tapetum; Te, tetrads; V, vacuole. Scale bars: B-J (10 µm); K-O (500 nm).

In *Arabidopsis*, three further maternal sporophytic cell layers surround the tapetum: the middle layer, the endothecium and the epidermis (Fig. 1A). The middle layer lies just outside of the tapetum and its function in pollen development remains unclear. The middle layer is surrounded by the endothecium layer, which ensures release of the mature pollen grains into the environment by enabling anther rupture after pollen maturation (Bonner and Dickinson, 1989). Finally, the external epidermal layer, covered with a functional cuticle, protects the anther itself from environmental stresses (Cheng and Walden, 2005).

Throughout the pollen development, the tapetum secretes a plethora of highly specific enzymes and metabolites involved in pollen grain formation, including callase, which mediates microspore release from tetrads (Bucciaglia and Smith, 1994), pollen coat proteins and other pollen coat components (Rejón et al., 2016). It is not known how these molecules remain corralled within the zone of active pollen development, and are prevented from diffusing into surrounding cell layers.

The ability of metabolites and proteins to move symplastically between cells depends on the presence of plasmodesmata, which allow continuity between the adjacent cytoplasms. In *Lilium* anthers, the tapetum and its neighbouring middle layer 1 have been reported to be symplastically isolated from each other and from other sporophytic cell layers (Clément and Audran, 1995). Thus, molecular movement between the tapetum and the middle layer involves traversing the apoplastic space.

Intriguingly, transmission electron microscopy studies at the end of the 20th century established that an enigmatic electron-dense apoplastic structure exists between the tapetum cells and the middle layer cells. This has been observed in several non-model plant species, including the gymnosperm *Pinus banksiana* (Dickinson, 1970), the monocot *Lilium* (Reznickova and Willemse, 1980) and several dicots (Galati et al., 2007; Heslop-Harrison, 1969; Platt et al., 1998; Staiger et al., 1994). This structure was named the peritapetal membrane (Dickinson, 1970) or the peritapetal wall (Reznickova and Willemse, 1980). Here, we have renamed this structure the peritapetal strip (PTS), because we feel that both the terms ‘membrane’ and ‘wall’ are misleading. The PTS was first observed during meiosis, persisting throughout subsequent pollen development (Dickinson, 1970; Reznickova and Willemse, 1980), and was proposed to contain pollen wall material (sporopollenin) owing to similarities in electron density and to resistance to acetolysis (Dickinson, 1970; Dickinson and Bell, 1972; Heslop-Harrison, 1969; Reznickova and Willemse, 1980). Based purely on the microscopical observations, these studies were not able to determine the function or properties of this structure.

Here, we provide evidence that pollen maturation in *Arabidopsis* is accompanied not only by symplastic isolation of the ZPD from external maternal tissues, but also by the gating of the apoplast between the ZPD and the middle layer. Using apoplastic fluorescent proteins, we observe the presence of an apparent apoplastic barrier between the two zones. We show that the formation of a functional PTS requires the NADPH oxidases RBOHE and RBOHC. Abnormal PTS development in the *rbohe rbohc* double mutant correlates with defective selective barrier function at the tapetum/middle layer boundary. An investigation of the composition of this structure leads us to conclude that it may comprise phenylpropanoid-containing polymers, including, as previously proposed, sporopollenin.

## RESULTS

### In *Arabidopsis*, the inner and outer anther cell layers are separated by a peritapetal strip

**Fig. 2.**
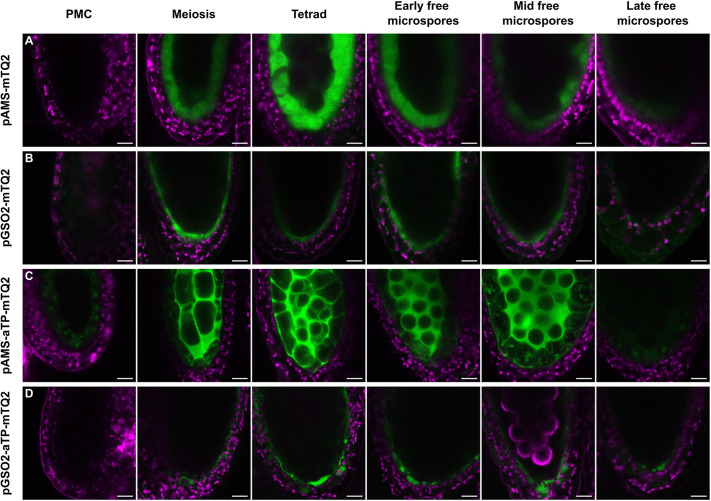
**Movement of free cytoplasmic mTQ2 and an apoplastically localised mTQ2 in *Arabidopsis thaliana* anthers.** (A) Free cytoplasmic mTQ2 expressed under the tapetum-specific *pAMS* in anthers. (B) Free cytoplasmic mTQ2 expressed under the middle layer-specific *pGSO2* in anthers. (C) The mTQ2 with the apoplast localisation signal aTP expressed under the tapetum-specific *pAMS* in anthers. (D) The mTQ2 with the apoplast localisation signal aTP expressed under the middle layer-specific *pGSO2* in anthers. PMC, pollen mother cell stage of pollen development. Scale bars: 10 µm.

Live anthers can be examined using confocal microscopy and fluorescent signals from the internal cell layers can be observed successfully (for example, see Fig. 2). Nevertheless, weaker signals are often masked by the autofluorescence of the surrounding tissues. We overcame this limitation by fixing the anthers and making them transparent with the ClearSeeAlpha optical clearing method (Kurihara et al., 2021; Ursache et al., 2018).

In cleared anthers at various stages of development, we observed a thin fluorescent line between tapetum and middle layer cells when the samples were excited with a UV laser (405 nm excitation) (Fig. 1B). By performing *z*-stack acquisition and 3D rendering, we could observe that this line is part of a three-dimensional structure that is reinforced at the external junctions between tapetum cells (Fig. 1C,D). This structure was also fluorescent when excited at 488 nm, 512 nm and 552 nm (Fig. 1E-G). It was first visible at the stage of meiosis and remained visible up to the late free-microspore stage (Fig. S1), which coincides with the degradation of the tapetum and middle layer cells (Quilichini et al., 2014).

To understand where this structure is located, we used the SCRI Renaissance Stain 2200, which stains the cell walls of all anther cell layers except the tapetum (Matsuo et al., 2013). This allowed us to confirm localisation to the interface between the tapetum and the middle layer (Fig. 1H-J).

Transmission electron microscopy (TEM) confirmed the appearance of a thin, continuous, electron-dense structure within the cell wall between the tapetum and the middle layer cells from meiosis onwards (Fig. 1K-O). Consistent with fluorescent staining, this structure appeared thicker, darker, and invaginated at the tapetum cell junctions (Fig. 1L-N).

Overall, this structure appeared similar to the peritapetal membrane observed in other plant species (Dickinson, 1970; Reznickova and Willemse, 1980). Here, we have renamed this structure the peritapetal strip (PTS). Although fluorescence staining did not always give the impression that the structure is fully continuous at all developmental stages, no discontinuities were observed using TEM (Fig. 1K-O). Discontinuities in fluorescence signal could therefore suggest a heterogeneity in the chemical composition of this structure (Fig. 1D). Thus, in *Arabidopsis thaliana*, the ZPD is separated from outer anther cell layers by a continuous apoplastic structure from the onset of meiosis.

### Symplastic movement between the tapetum and the middle layer is reduced or lost from meiosis onwards

The presence of a PTS prompted us first to investigate connectivity in the maternal anther tissues. Reports have suggested that cytoplasmic connections are lost not only between tapetal cells and pollen precursors, but also between tapetum cells and the middle layer following meiosis (Clément and Audran, 1995; Owen and Makaroff, 1995). Free, cytoplasmic green fluorescent protein (GFP) is a small molecule (27 kDa) that can move between symplastically connected cells allowing a non-invasive approach to assess the status of symplastic cellular connections (Stadler et al., 2005). We used a GFP variant, mTurquoise2 (mTQ2), which retains its fluorescent properties in various pH conditions and is thus suitable for imaging in both the symplast and apoplast (Stoddard and Rolland, 2019). Free mTQ2 expression was driven by the *AMS* promoter, which is active in tapetum cells from meiosis onwards (Truskina et al., 2022). mTQ2 signal was detected exclusively in the tapetum cells from meiosis onwards (Fig. 2A), indicating that this protein cannot freely diffuse from the tapetum to the middle layer. Similarly, when we expressed the free mTQ2 in the middle layer under the *GSO2* promoter, which is active in the middle layer prior to the onset of meiosis and up to the point of tapetum degeneration (Truskina et al., 2022), no fluorescent signal was detected in the tapetum (Fig. 2B), although fluorescence was detected in the endothecium and epidermis. Our results suggest that symplastic movement between the middle layer and the tapetum is reduced or lost from meiosis onwards.

### The PTS colocalises with a functional apoplastic barrier

The symplastic isolation of the tapetum from outer cell layers implies that all molecules entering the ZPD from the middle layer must traverse the apoplast and thus the PTS. To evaluate whether the PTS affects molecular movement across this apoplastic interface, we investigated the diffusion of an apoplastically targeted mTQ2, produced by fusion to the apoplast targeting sequence (aTP) of the *Arabidopsis thaliana* 2S2 protein (Sahoo et al., 2014). The resulting aTP-mTQ2 protein was expressed in the tapetum using *pAMS*. The aTP-mTQ2 fusion protein was able to freely diffuse into the locular matrix and in the apoplast around the tapetum cells, but was never observed around the middle layer cells (Fig. 2C). To test movement in the opposite direction, we expressed the aTP-mTQ2 protein fusion in the middle layer under the *GSO2* promoter. In these lines, aTP-mTQ2 was detected strongly in the cells of the middle layer, but also in endothecium and epidermis cells. Some apoplastic signal was also detected around these cells, but no signal was detected in the locular matrix or around the tapetum cells (Fig. 2D). The apparently poor secretion of aTP-mTQ2 by middle layer cells compared with the tapetum, may be a consequence of their different biological functions, and makes interpretation of *pGSO2-aTP-mTQ2* lines complex. Nonetheless, our experiments suggest that the diffusion of apoplastic proteins is restricted at the middle layer–tapetum interface, supporting the hypothesis that the PTS may prevent diffusion of larger molecules (such as fluorescent proteins) between the ZPD and surrounding sporophytic tissues.

### The establishment of the PTS requires the NADPH oxidases RBOHE and RBOHC

The plant-specific RBOH family proteins are plasma membrane-localised NADPH oxidases. When activated, they produce O_2_^−^ in the apoplast, which is then converted into H_2_O_2_ that can be harnessed by apoplastic peroxidases to fuel reactive oxygen species (ROS)-dependant reactions, including lignin polymerisation (Lee et al., 2013; Marino et al., 2012; Suzuki et al., 2011). Among the ten RBOH-encoding genes in *Arabidopsis thaliana*, *RBOHE* and *RBOHC* have previously been proposed to be important for tapetum programmed cell death at the later stages of pollen development (Xie et al., 2014). However, *RBOHE* and *RBOHC* are expressed early in the tapetum, from the onset of the meiosis (Xie et al., 2014) (Fig. S2A). Thus, it is possible that these enzymes could have other functions during earlier stages of anther development.

**Fig. 3.**
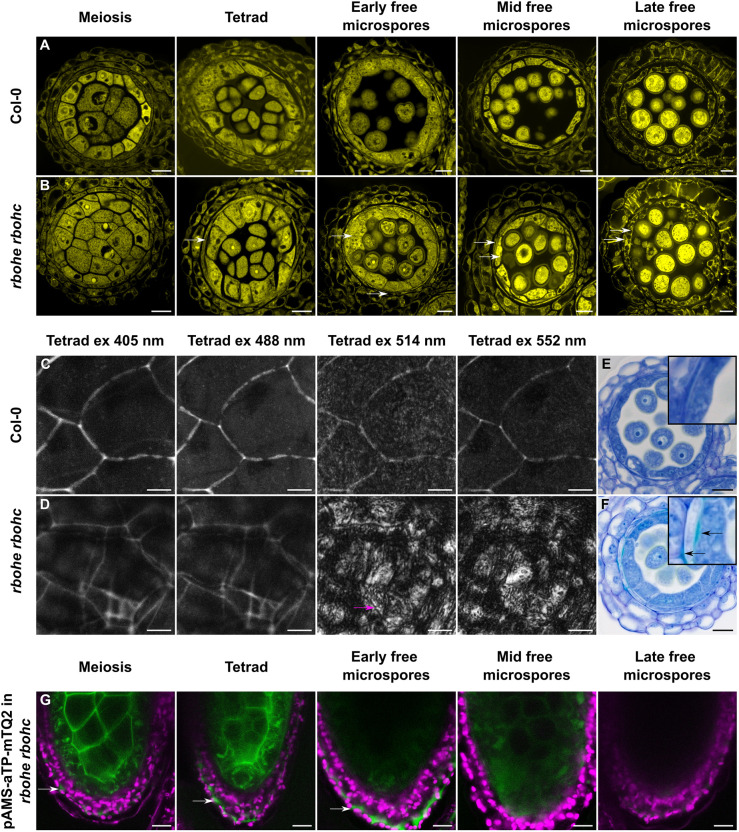
**The PTS is structurally and functionally defective in the *rbohe rbohc* double mutant.** (A,B) Pollen and anther development in the wild type (A) and the *rbohe rbohc* double mutant (B). Arrows indicate hypertrophied middle layer and hypertrophied tapetum in the mutant. (C,D) The PTS in the wild type (C) and the *rbohe rbohc* double mutant (D) at the tetrad stage of anther development visualised at different excitation (ex) wavelengths. Arrow indicates ‘detached’ signal in the mutant. (E,F) Ectopic lignin-like deposition around the middle layer in the *rbohe rbohc* double mutant (F, arrows) compared with the wild type (E) visualised using Toluidine Blue staining. Insets show higher magnification images of E and F. (G) mTQ2 with the apoplast localisation signal aTP expressed under the tapetum-specific *pAMS* in anthers. Arrows indicate mTQ2 signal around endothecium and epidermis cells in the *rbohe rbohc* double mutant. Scale bars: 10 µm (A,B,E-G); 5 µm (C,D).

In line with this, an in-depth analysis of the previously described *rbohe rbohc* double mutant revealed several defects during early stages of anther development. Following the release of microspores from tetrads, both the tapetum and the middle layer cells become swollen (hypertrophied) and irregularly shaped (Fig. 3A,B) in this background. When we imaged the *rbohe rbohc* PTS at 512 or 552 nm, the PTS signal was abnormally diffuse and appeared to be excluded from tapetal cell boundaries (Fig. 3C,D). However, when imaged at 405 or 488 nm, the PTS signal was not diffuse, but was weaker and more continuous than that observed in wild-type anthers, and appeared to be composed of two layers (Fig. 3C,D). The origin of this ‘double’ PTS signal became clear when we studied cross-sections stained with Toluidine Blue, which stains lignin-like compounds a blue-green colour (O'Brien et al., 1964). In the *rbohe rbohc* double mutant, the apoplastic spaces between the tapetum and the middle layer as well as between the middle layer and the endothecium were filled with deposits of ectopic lignin-like material (Fig. 3E,F). Thus, the ‘double’ PTS signal appears to originate from an ectopic lignification around the middle layer cells. The defects in the PTS of the *rbohe rbohc* double mutant first appeared at meiosis, prior to the visible hypertrophy of the middle layer and tapetum (Fig. S3). The discrepancy between the intensity of PTS fluorescence (weaker in *rbohe rbohc* double mutants than in wild type, at least at early stages) and Toluidine Blue staining (strong, turquoise lignin-associated staining in *rbohe rbohc* double mutants compared with no visible staining in wild-type anthers) could suggest that the chemical composition of the fluorescent cell wall material in these two backgrounds is different.

To analyse the functionality of the PTS, we expressed the apoplastically targeted aTP-mTQ2 protein in the tapetum under the *pAMS* promoter in the *rbohe rbohc* double mutant. The *AMS* promoter was expressed normally in this background (Fig. S4). In contrast to wild-type anthers in which the aTP-mTQ2 signal was restricted to the ZPD (Fig. 2C), in the *rbohe rbohc* double mutant the aTP-mTQ2 signal was additionally detected in the apoplastic spaces surrounding the middle layer, endothecium and epidermis (Fig. 3G). Thus, aTP-mTQ2 is able to traverse the PTS in *rbohe rbohc* double mutants, indicating defects in the blockage of apoplastic diffusion.

When aTP-mTQ2 was expressed in the middle layer using pGSO2 in the *rbohe rbohc* double mutant background, as in wild-type plants the aTP-mTQ2 signal was observed in the middle layer, endothecium and epidermis, predominantly within cells. The GSO2 promoter was expressed normally in this background (Fig. S4). However, no obvious signal was observed in the tapetum or locular matrix (Fig. S2B). This apparently contradictory result may be due to the relatively low activity of the *GSO2* promoter compared with that of *AMS*, or, as discussed above the apparent lack of strong secretion of aTP-mTQ2 from middle layer cells, combined with the technical constraints of observing low quantities of mTQ2 signal within the extensive apoplast of the ZPD.

### The PTS is likely to contain phenylpropanoid-pathway derived compounds

The RBOH NADPH oxidases are known to produce ROS required for lignin polymerisation during Casparian strip formation in the root endodermis (Fujita et al., 2020; Lee et al., 2013). The presence of a defective PTS in the *rbohe rbohc* double mutant as well as the fluorescent properties of this structure therefore prompted us to investigate whether the PTS could be composed of lignin or lignin-like materials.

Apoplastic polymer composition can, to a certain extent, be inferred using histochemical stains (Ursache et al., 2018). Thus, to assess the composition of the PTS, we tested a variety of ClearSee-compatible stains for their ability to stain the PTS, and compared this with their ability to stain other structures in the anthers, such as the anther xylem (composed of lignin), pollen wall (composed of sporopollenin), mature endothecium (containing lignin) and anther epidermis (containing cutin) (Fig. S5). We found that the PTS could be stained with Auramine-O and Basic Fuchsin, relatively non-specific dyes that also stain lignin-containing xylem and mature endothecium, the epidermal cuticle and the sporopollenin-containing pollen wall. In contrast, berberine chloride and berberine hemisulphate stained the PTS only weakly. These dyes also stained the lignin-containing xylem and the lignified endothecium, but not the sporopollenin of the pollen wall or the cuticle. Finally, the lipophilic dye Nile Red was only able to stain the epidermal cuticle, but did not stain the PTS, pollen wall, xylem or lignified endodermis (Fig. S5). Taken together with the fact that the PTS appears well before the initiation of sporopollenin biosynthesis, these experiments suggest that the early PTS may primarily consist of phenolic metabolites resembling lignin. This possibility is also supported by the high intensity of emissions and the broad range of excitation/emission spectra (Donaldson, 2020).

**Fig. 4.**
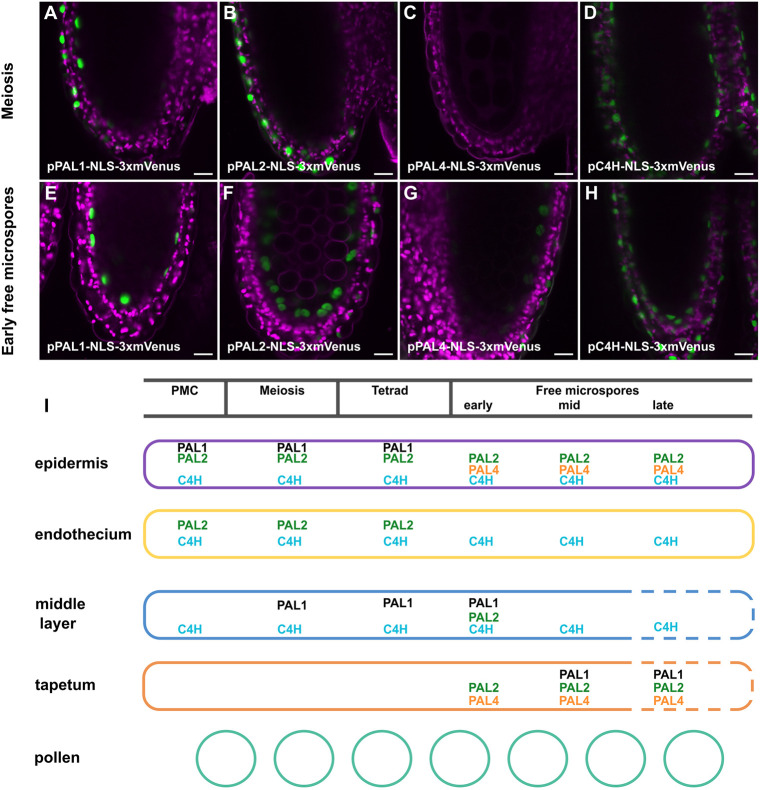
**Expression of genes encoding components of the phenylpropanoid biosynthesis pathway in anthers.** (A-H) *PAL1* (A,E), *PAL2* (B,F), *PAL4* (C,G) and *C4H* (D,H) expression at the meiosis and early free-microspore stages. (I) Schematic illustrating expression of genes involved in the phenylpropanoid biosynthesis pathway in anthers at different stages of pollen development (see Fig. S6). Scale bars: 10 µm.

Phenolic compounds, are synthesised via the phenylpropanoid pathway from phenylalanine by the consecutive action of the PHENYLALANINE AMMONIA-LYASE (PAL) and CINNAMATE 4-HYDROXYLASE (C4H) enzymes. The biosynthesis of most, but not all, lignin monomers requires further lignin-specific enzymes, such as the CINNAMOYL-COA REDUCTASE (CCR) (Vanholme et al., 2019). In *Arabidopsis thaliana*, there are four PAL-encoding genes (*PAL1* to *PAL4*), a single *C4H* gene and two CCR-encoding gene homologues (*CCR1* and *CCR2*) (Fraser and Chapple, 2011; Vogt, 2010). To further support the hypothesis that the PTS is made from phenolics, we analysed the expression of some of these genes using promoter expression lines containing 3xmVenus reporter lines (Fig. 4, Fig. S6) (Andersen et al., 2021). We found that *PAL1*, *PAL2* and *PAL4* are expressed in the epidermis throughout anther development, in the middle layer and the endothecium from the pollen mother cell stage up to the release of the microspores from the tetrads, and in the tapetum from the free-microspore stages onwards (Fig. 4, Fig. S6). *PAL3* expression was not detected in anthers (Fig. S6). *C4H* was expressed in the epidermis, endothecium and middle layer at all stages of the anther development. Although we were not able to detect strong *CH4* expression in the tapetum (Fig. 4, Fig. S6), some expression has been observed from stage 8 (microspore release) onwards in another study (Xue et al., 2020). However, because the PTS is observed from much earlier in development our data suggest that the phenolic precursors integrated within PTS at the meiosis stage are likely to be synthesised in the middle layer.

**Fig. 5.**
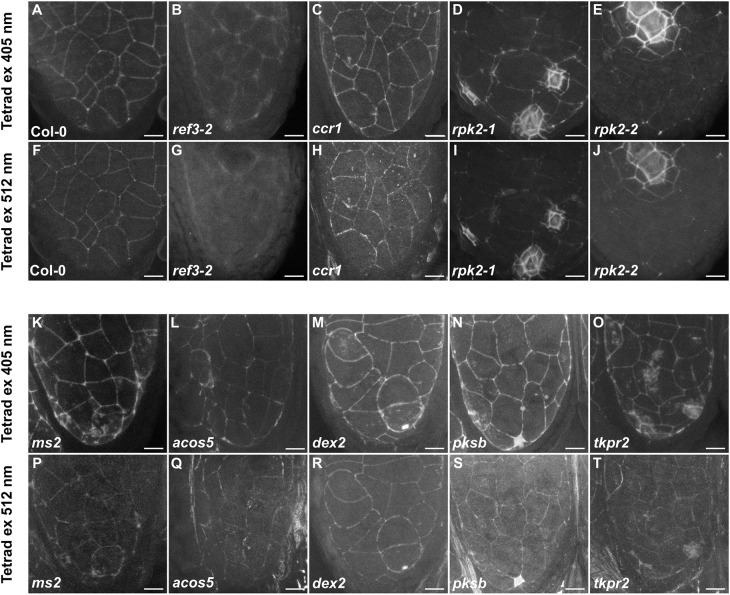
**Characterisation of the PTS in mutants defective in the phenylpropanoid biosynthesis pathway, in the *rpk2* mutants and in sporopollenin biosynthesis pathway mutants.** (A-T) The PTS in the wild type, Col-0 (A,F), and in *ref3-2* (B,G), *ccr1* (C,H), *rpk2-1* (D,I), *rpk2-2* (E,J), *ms2* (K,P), *acos5* (L,Q), *dex2* (M,R), *pksb* (N,S) and *tkpr2* (O,T) mutants at the tetrad stage using either 405 nm or 512 nm excitation (ex) wavelengths. Scale bars: 10 µm.

The importance of the phenylpropanoid pathway for the PTS formation was further assessed using mutants of the C4H gene, *ref3-2* and *ref3-1.* These missense mutants do not completely abolish the activity of the phenylpropanoid pathway, but lead to reduced content of lignin and potentially other phenolics (Schilmiller et al., 2009). The *ref3-1* mutant, which is considered to be phenotypically less severe than *ref3-2*, is in the Ler background (Schilmiller et al., 2009); no major differences in the PTS in Ler wild-type plants was detected compared with the Col-0 wild type (Figs S1, S7). The stronger *ref3-2* mutant produced a uniformly weak and diffuse fluorescent PTS signal visible using 405 nm and 488 nm excitation wavelengths, although when excited at 514 nm and 552 nm the signal was almost absent (Fig. 5B,G, Fig. S8). The *ref3-1* mutant also produced a very weak signal (Fig. S9). These observations strongly suggest that metabolites from the phenylpropanoid pathway are necessary for the formation of a functional PTS.

The expression of the lignin biosynthesis genes *CCR1* and *CCR2* (at lower levels) has been detected in the tapetum-specific transcriptome (Li et al., 2017b). Owing to our inability to produce a flowering *ccr1 ccr2* double mutant in our growth conditions, PTS formation was only assessed in the *ccr1* single mutant (Fig. 5C,H; Fig. S10). *ccr1* single mutants exhibit reduced endothecium lignification, collapsed xylem vessels and reduced lignin content in stems (Thévenin et al., 2011). Our experiments revealed a defect in the PTS at the meiosis stage, with the PTS only being visible when excited by 405 nm and 488 nm wavelengths. At the later stages, the PTS appeared to be more discontinuous, and to contain diffuse ‘patches’, particularly when excited at 514 nm and 552 (Fig. S10). These results might indicate the presence of a partially defective PTS, suggesting potential involvement of CCR1-catalysed lignin in its formation.

The potential importance of the middle layer in the biosynthesis of phenolic compounds prompted us to investigate the presence of the PTS in plants lacking RECEPTOR-LIKE PROTEIN KINASE 2 (RPK2), which have previously been reported to lack the correctly specified middle layer, produce a hypertrophied tapetum, and show an inadequate lignification of the endothecium (Cui et al., 2018; Mizuno et al., 2007). Our histological sections confirmed that loss of *rpk2* function causes defects in the formation of the middle layer (Figs S11 and S12). Our data suggest, as previously proposed, that rather than ‘lacking’ the middle layer, in *rpk2* mutants a ‘hybrid’ cell layer with characteristics of both the endothecium and middle layer is formed (Fig. S12G-J). The PTS signal in the *rpk2* mutants *rpk2-1* and *rpk2-2* appears to be reinforced by ectopic lignin-like material deposited around the entire periphery of these ‘hybrid’ cells adjacent to the tapetum (Fig. 5D,E,I,J; Figs S11, S12). It is possible that, in the absence of the correctly specified middle layer, PTS defects lead to the compensatory deposition of ectopic lignin-like material as seen in the *rbohe rbohc* double mutant. Indeed, blue-green Toluidine Blue-stained lignin was detected in the cross-sections of the *rpk2-2* mutant (Fig. S12E,F). Thus, the middle layer appears to be particularly important for PTS formation and might contribute to its composition by supplying specific phenolic compounds.

Besides lignin, as suggested above and proposed previously, sporopollenin could be a PTS component. This anther-specific polymer is produced in the tapetum from the tetrad stage onwards and is then secreted into the locular matrix before assembling on the pollen surface. Multiple enzymes participate in sporopollenin biosynthesis, including MALE STERILITY 2 (MS2), POLYKETIDE SYNTHASES A and B (PKSA/PKSB), TETRAKETIDE ALPHA-PYRONE REDUCTASES 1 and 2 (TKPR1/TKPR2), ACYL-COA SYNTHETASE 5 (ACOS5) and CYTOCHROME P450 enzymes CYP703A2 and CYP704B1 (Quilichini et al., 2015). Given that sporopollenin biosynthesis initiates only during microspore release, it is likely that sporopollenin is not a component of the early PTS but might reinforce it later in development. To test this, we analysed the PTS in the sporopollenin biosynthesis mutants *ms2*, *acos5*, *dex2* (which lacks CYP703A2), *pksb* and *tkpr2*. The *ms2* mutant is in the Ler background (Aarts et al., 1997); the PTS in the Ler wild-type background strongly resembles that in the Col-0 wild-type background (Figs S1, S7). In the *ms2* mutant, the PTS appeared intact but contained additional patchy signals not observed in the wild-type anthers (Fig. 5K,P; Fig. S13). In the *acos5* mutant, additional patchy signals at the free-microspore stages were also observed but were not as frequent as in the *ms2* mutant (Fig. 5L,Q, Fig. S14). In the *dex2* mutant, the PTS appeared similar to the wild type (Fig. 5M,R, Fig. S15). In the *pksa* and *tkpr2* mutants, we could occasionally observe large spots of strong fluoresce at the PTS (Fig. 5N,S,O,T; Figs S16, S17). The origin of the patches that appear in these mutants is unclear, but could indicate the presence of a compensatory mechanism triggered by failures in the selective barrier function of the PTS. It should be borne in mind that the single *pksa* and *tpkr2* mutants analysed here do not completely abolish sporopollenin biosynthesis because the affected genes act redundantly with their paralogues *PKSB* and *TKPR1*, respectively (Quilichini et al., 2015).

## DISCUSSION

One of the distinguishing features of the embryophytes is that haploid reproductive cells develop enclosed within sterile multicellular structures (Niklas and Kutschera, 2010). This feature ensure protection of the developing (macro- or micro-) spores from the environmental stresses associated with terrestrialisation. These sterile cell layers not only became important in the process of transferring nutrients to developing spores, but acquired specialised metabolic functions supporting spore development. In the male reproductive structures of higher plants, the tapetum cell layer is the predominant source of highly specific metabolites, including pollen wall and pollen coat constituents, enzymes and other proteins necessary for the development of the adjacent pollen grains (Bucciaglia and Smith, 1994; Gómez et al., 2015; Pacini et al., 1985; Quilichini et al., 2015; Rejón et al., 2016). The presence of a highly metabolically active tapetum secreting specialised metabolites may therefore have necessitated reinforced control of molecular movement. Consistent with this idea, we here provide strong evidence for the previously suggested symplastic isolation of the tapetum and the developing pollen from the other sporophytic tissues. In addition, we demonstrate the presence of an apoplastic selective barrier, the PTS, which gates apoplastic diffusion between the tapetum and the outer cell layers of the anther. Our data support our proposition that the tapetum and the developing pollen grains (microspores) constitute a metabolically contained unit termed the ZPD.

The PTS is one of only a few apoplastic barriers currently described in plants. Others include the Casparian strip, which surrounds the root endodermis and isolates the stele apoplast from that of the root cortex, the cuticle on the surface of the aerial organs and the developing embryo, suberised layers found within the seed coat, and the pollen wall on the surface of pollen grains (Nawrath et al., 2013). These selective barriers are composed of diverse polymers: endodermis-residing barriers are initially composed of lignin and are later reinforced with entire surface-spanning suberin deposits, the cuticle consists of cutin (a complex matrix of aliphatic and phenolic components) and waxes, and the pollen wall contains sporopollenin. These differences may reflect the different permeabilities of each selective barrier. Based on its position, the PTS must permit the diffusion of nutrients, such as sugars and amino acids, from the mother plant to the developing pollen. In addition, our recent work suggests that the PTS is permeable to small peptides that are perceived in the middle layer and coordinate tapetum and pollen grain development (Truskina et al., 2022). Our current data suggest that the PTS serves to impede the movement of larger molecules, such as proteins, possibly acting as a size- and/or charge-specific filter. Nonetheless, analysing the precise biophysical and chemical properties of this filter promises to present a significant technical challenge owing to the localisation and extreme thinness of the PTS. Permeability assays to test the capacity of molecules (particularly dyes) with varying sizes and properties to diffuse across the PTS have proved challenging owing to difficulty in imaging internal tissues when the external epidermis layer contains cuticle that hinders dye diffusion towards inner cell layers and causes variability in dye penetration. However, optimisation of such assays will undoubtedly be a goal in future studies.

Our results show the requirement of RBOH proteins for PTS integrity and functionality, supporting the idea that a ROS-mediated polymerisation reaction could occur during PTS formation. Consistent with this finding, we also provide data to support the idea that the PTS contains lignin-like phenolic compounds (Tobimatsu and Schuetz, 2019). The defective PTSs observed in mutants with reduced C4H function strongly suggest the involvement of the phenylpropanoid pathway in PTS formation. The exact composition of the PTS nonetheless remains unknown. One of indicators classically used to show the presence of lignin is turquoise staining by the polychromatic stain Toluidine Blue. Although this is observed for the ectopic lignification observed in the *rbohe rbohc* and *rpk2* mutants, no such staining is visible at the PTS of wild-type plants. However, as the wild-type PTS is extremely thin, it may be difficult to visualise using this stain. In conclusion, further investigations will be required to understand the proposed contribution of a lignin-like polymer to PTS construction.

Although our data suggest that suberin and cutin can be excluded as potential polymers within the PTS based on the lack of staining by the lypophilic stain Nile Red (Fig. S5) (Ursache et al., 2018), the juxtaposition of the PTS with the tapetum prompted us to investigate sporopollenin as a potential PTS constituent. The expression of most of the genes known to be involved in sporopollenin biosynthesis initiates in the tapetum at the tetrad stage, later than the initiation of PTS formation. We thus considered that sporopollenin might reinforce the PTS at later stages of pollen development. This appears to be plausible as the anther continues to grow throughout pollen development, necessitating continuous PTS reinforcement. Fragmentation of an early PTS containing lignin-like molecules, and reinforcement with compositionally distinct polymer (such as sporopollenin) would provide an explanation for the rather discontinuous fluorescent signal that we observe in the wild-type PTS, despite the apparent continuity of the PTS using TEM-based techniques. Furthermore, the defects we observed in the PTS of some mutants defective in sporopollenin biosynthesis, including the apparent deposition of ectopic PTS material, support the idea that the PTS of these mutants may be functionally affected, triggering compensatory mechanisms.

In both the *rbohe rbohc* double mutant and the *rpk2* mutants the apparent defects in the PTS are associated with an over-lignification around the middle layer cells, expanding the normal domain of the PTS to the middle layer/endothecium interface (Figs 3, 5, Figs S3, S11, S12). Despite this, our results suggest that the PTS remains functionally compromised, at least in *rbohe rbohc* double mutants. This situation is very similar to that seen in mutants defective in the formation of the Casparian strip, which also undergo compensatory ectopic lignification and suberisation but remain functionally defective (Hosmani et al., 2013; Kalmbach et al., 2017; Kamiya et al., 2015; Li et al., 2017a). Furthermore, compensatory lignin in the root endodermis has been shown to differ, in terms of composition, from Casparian-strip lignin (Reyt et al., 2021).

Intriguingly, in the root endodermis, the localised lignin polymerisation required for Casparian strip integrity is achieved through the highly localised activation of RBOH proteins situated adjacent to gaps in the barrier via the SHENGEN (SGN) integrity monitoring pathway (Fujita et al., 2020; Lee et al., 2013). Lignin monomers involved in this process are also thought to be produced by the endodermis (Andersen et al., 2021). By contrast, our data suggest that the ROS necessary for early PTS formation may originate in the tapetum, whereas phenolic PTS components are produced, at least initially, by the middle layer. Limited diffusion of both ROS and phenolic compounds within the cell wall could provide an elegant mechanism for ensuring the inter-layer positioning of the nascent PTS. Furthermore, our finding that mutants lacking intact PTSs undergo apparently compensatory deposition of lignin, strongly suggests that, as is the case in the Casparian strip, the integrity of the PTS may be actively monitored. It is tempting to suggest that similar peptide-mediated monitoring mechanisms may be involved in both systems, particularly in light of the recent finding that the receptor kinases GASSHO1 (GSO1) (also known as SGN3) and GSO2 coordinate tapetum activity with pollen grain development through their activity in the middle layer (Truskina et al., 2022). However, this possibility requires further investigation.

## MATERIALS AND METHODS

### Plant materials and growth conditions

Seeds were sown on soil, stratified for 2 days at 4°C and grown in long-day conditions (16 h light/8 h dark). Some transgenic plants were examined in the T1 generation; these were first selected on half-strength MS medium with 1% sucrose and 1% agar supplemented with either 50 µg/ml kanamycin or 10 µg/ml glufosinate ammonium (Basta).

Mutant alleles used were: *rbohe-2 rbohc* (*rbohe-2 rhd2-1*) (Xie et al., 2014), *rpk2-1* (SALK_062412) (Mizuno et al., 2007), *rpk2-2* (SALK_039514) (Mizuno et al., 2007), *ref3-1*, *ref3-2* (Schilmiller et al., 2009), *ms2* (Aarts et al., 1997), *ccr1-3* (SALK_123689, *ccr1s*) (Mir Derikvand et al., 2008; Panda et al., 2020), *acos5* (SK19167), *dex2-2* (SALK_119582) (Morant et al., 2007), *pksb* (GABI_454C04) (Kim et al., 2010) and *tkpr2-1* (SALK_129453) (Grienenberger et al., 2010). The genotyping primers are listed in Table S1. The *pPAL1-NLS-3xmVenus*, *pPAL2-NLS-3xmVenus*, *pPAL4-NLS-3xmVenus*, *pPAL4-NLS-3xmVenus* and *pC4H-NLS-3xmVenus* lines are described by Andersen et al. (2021). The *pAMS-NLS-3xmVenus* and *pGSO2-NLS-3xmVenus* reporter lines are described by Truskina et al. (2022).

### Generation of transgenic plant lines

Gateway MultiSite cloning was used to generate transgenic lines.

For the *pRBOHE-NLS-3xmVenus* transcriptional reporter line, the 4105 bp promoter of *RBOHE* from −4105 bp to −1 bp was amplified by PCR from *Arabidopsis* (Col-0) genomic DNA, inserted into pDONR P4-P1R and recombined with 3xmVenus-N7 pDONR211, OCS terminator pDONR P2R-P3 (containing STOP codon followed by the octopine synthase terminator) and pK7m34 GW destination vector (with kanamycin *in planta* resistance), and transformed into Col-0 plants.

To create *pAMS-aTP-mTQ2* and *pGSO2-aTP-mTQ2* lines, the open reading frame (ORF) encoding the aTP of the *Arabidopsis thaliana* 2S2 protein (Sahoo et al., 2014) and the *mTQ2* ORF sequence lacking the start codon but including the stop codon were separately amplified by PCR and then combined using overlap extension PCR. The resulting *aTP-mTQ2* was inserted into pDONR211 and then recombined with the *pAMS* pDONR P4-P1R or *pGSO2* pDONR P4-P1R, OCS terminator pDONR P2R-P3 and pB7m24GW,3 destination vector (with Basta *in planta* resistance). The resulting constructs were transformed into Col-0 or *rbohe rbohc* double-mutant plants.

To create *pAMS-mTQ2* and *pGSO2-mTQ2* reporter lines, the *mTQ2* ORF containing the start codon and the stop codon was amplified by PCR, inserted into pDONR211 and then recombined with the *pAMS* pDONR P4-P1R or *pGSO2* pDONR P4-P1R, OCS terminator pDONR P2R-P3 and pB7m24GW,3 destination vector (with Basta *in planta* resistance). The resulting constructs were transformed into Col-0 plants.

To verify expression of *pAMS* and *pGSO2* in the *rbohe rbohc* mutant background, reporter constructs *pAM3-NLS-3xmVenus* and *pGSO2-NLS-3xmVenu*s (as previously described by Truskina et al., 2022) were transformed directly into Col-0 and *rbohe rbohc* double mutants.

The cloning primers are listed in Table S1.

### Histology

Inflorescences were fixed with FAA [50% (v/v) ethanol, 5% (v/v) acetic acid, 3.7% (v/v) formaldehyde] overnight, dehydrated in a graded series of 50%, 60%, 70%, 85%, 95% and 100% of ethanol for 1 h each, then further incubated overnight in 100% ethanol. The samples were then incubated in 50% ethanol/50% Technovit 7100 base liquid (v/v) for 4 h and then in 25% ethanol/75% Technovit 7100 base liquid (v/v) overnight. The samples were infiltrated in Technovit 7100 infiltration solution (1 g hardener I in 100 ml Technovit 7100 base liquid) with vacuum for 2 h and then incubated for 6 days. All steps above were conducted at room temperature (RT) with gentle agitation. The samples were polymerised with Technovit 7100 polymerisation solution (100 µl Technovit 7100 hardener II in 1.5 ml infiltration solution) at RT for 6 h. Transverse sections of 3 µm were cut using a Leica Microtome HM355S.

For histological analysis, the sections were stained with 0.01% (w/v) acriflavine in H_2_O for 5 min, mounted in VECTASHIELD (Vector Laboratories) and observed using a TCS SP5 confocal microscope (Leica) with excitation at 488 nm and emission at 492-551 nm.

Alternatively, the sections were stained with 0.05% (w/v) Toluidine Blue in H_2_O for 1 min, mounted in Entellan mounting medium (Sigma-Aldrich) and observed under a Zeiss Axio Imager M2 microscope.

### ClearSee tissue clearing

Inflorescences were fixed in 4% paraformaldehyde in PBS at 4°C under vacuum for 2 h and kept subsequently overnight at 4°C. The samples were washed twice with PBS and cleared with ClearSee Alpha solution [10% (w/v) xylitol powder, 15% (w/v) sodium deoxycholate, 25% (w/v) urea and 0.63% (w/v) sodium sulphite] for 1 week changing to a fresh solution every 2 days at RT.

Anthers were dissected from the inflorescences, mounted in ClearSee Alpha solution and observed under a confocal TCS SP8 confocal microscope (Leica) using a 40× oil objective. Autofluorescence was observed using 405 nm excitation with 413-467 nm emission, 488 nm excitation with 492-546 nm emission, 514 nm excitation with 516-570 nm emission, or 552 nm excitation with 555-609 nm emission.

Alternatively, the samples were stained overnight with either 0.1% (w/v) Auramine O in ClearSee Alpha, 0.2% (w/v) Basic Fuchsin in ClearSee Alpha, 0.05% (w/v) Nile Red in ClearSee Alpha, 0.1% (w/v) berberine chloride in ClearSee Alpha, 0.1% (w/v) berberine hemisulphate in ClearSee Alpha or 0.5% (v/v) SCRI Renaissance Stain 2200 in ClearSee Alpha. The samples were washed three times for 20 min each with ClearSee Alpha solution, dissected, mounted in ClearSee Alpha solution and observed under a confocal TCS SP8 confocal microscope (Leica) using a 40× oil objective. The excitation and emission wavelengths were as follows: Auramine-O excitation 488 nm, emission 500-570 nm; Basic Fuchsin and Nile Red excitation 552 nm, emission 556-631 nm; berberine chloride and berberine hemisulphate excitation 488 nm, emission 491-545 nm. For colocalisation microscopy, sequential scanning was used with the PTS visualised using 514 nm excitation with 516-570 nm emission, and the SCRI Renaissance Stain 2200 stain visualised using 405 nm excitation with 410-473 nm emission.

### TEM

Flower buds at appropriate developmental stages were fixed with 4% (w/v) formaldehyde and 2% (w/v) glutaraldehyde in 0.1 M phosphate buffer (pH 7.2) (PB) under vacuum (0.6 bar) at 4°C for 1 h during which the vacuum was slowly broken three times, then incubated in fresh fixative solution at 4°C overnight. The samples were washed three times in PB, postfixed for 2 h in 1% (w/v) osmium tetroxide in PB at RT, rinsed five times for 5 min in PB and dehydrated under vacuum in a graded ethanol series for 20 min each time, increasing in five steps from 30 to 100% at RT. The samples were then incubated in graded low-viscosity SPURR resin in ethanol (33%, 66% and twice 100%) at 4°C for 24 h each (including 20 min under vacuum). The samples were polymerised in fresh SPURR resin at 60°C for 18 h. Ultrathin sections (70 nm) were prepared using a UC7 Leica ultramicrotome, placed on formvar-coated grids, then poststained in 2% uranyl acetate and lead citrate. Sections were examined under a JEOL 1400 transmission electron microscope at 120 kV and imaged with a Gatan Rio 16 camera.

### Confocal microscopy

For the 3xmVenus and mTQ2 reporter lines, the anthers were stained with 20 μg/ml propidium iodide solution and examined using TCS SP5 (Leica) TCS SP8 (Leica) confocal microscopes using 40× oil objectives. The mVenus reporter lines were visualised using excitation at 514 nm and emission at 526-560 nm for mVenus or 605-745 nm for propidium iodide. The mTQ2 reporter lines were visualised by sequential excitation with 448 nm excitation and 452-505 nm emission for mTQ2, and 514 nm excitation and 632-726 nm for propidium iodide.

## Supplementary Material

10.1242/develop.200596_sup1 Supplementary information
