# Supplementary material for: Anther development in Arabidopsis thaliana involves symplastic isolation and apoplastic gating of the tapetum-middle layer interface
Source: Development. 2022 Nov 16;149(22):dev200596. doi: 10.1242/dev.200596 (PMC10114112; doi:10.1242/dev.200596)
Supplement: Supplementary information [file develop-149-200596-s1.pdf]

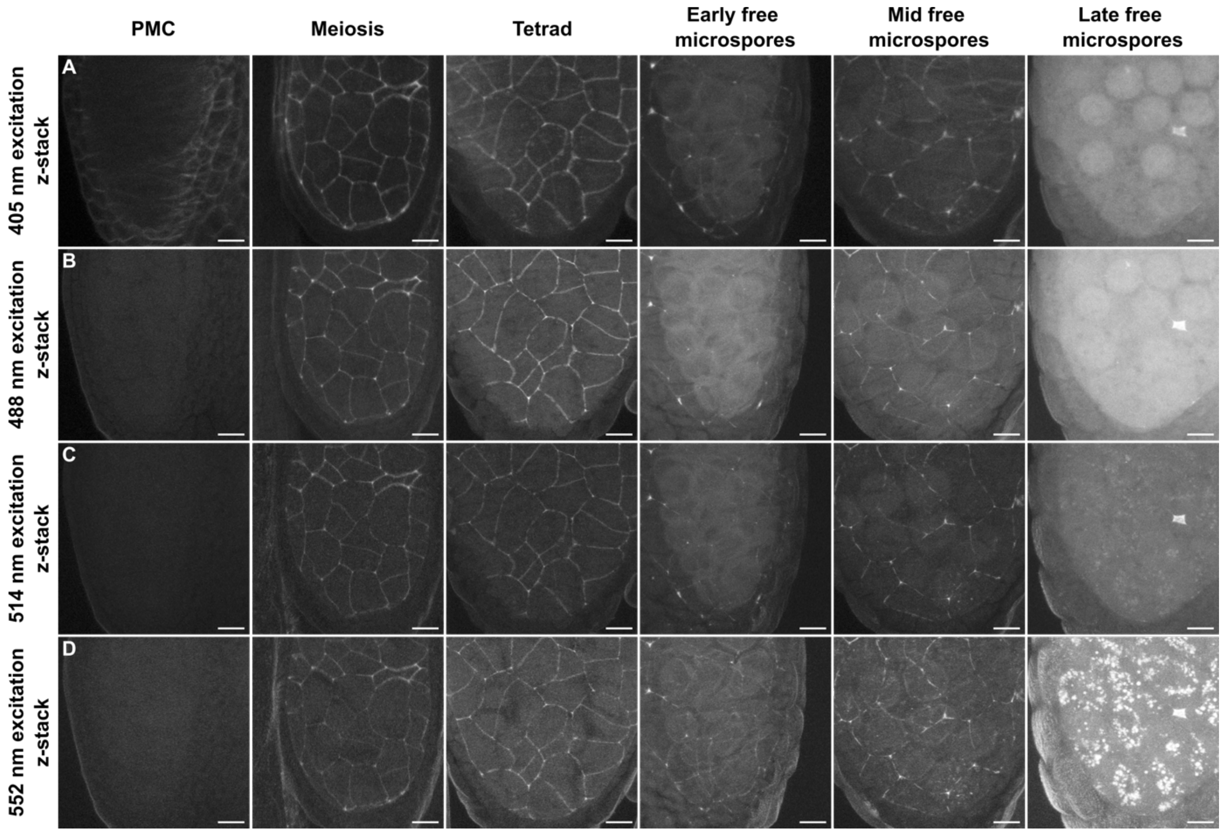

Fig. S1. Peritapetal strip in Col-0 wild-type anthers. The PTS was exited at 405 nm (A), 488 nm (B), 514 nm (C) and 552 nm (D). Scale bars: 10 µm

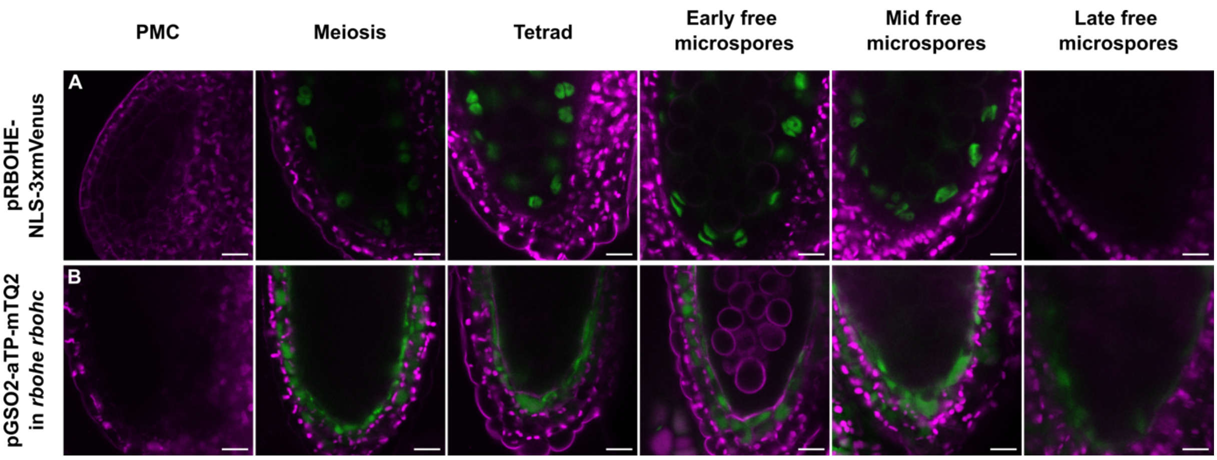

Fig. S2. Expression of the *RBOHE* gene in anthers using an *pRBOHE-NLS- 3xmVenus* reporter line (A) and the localization of the apoplastic mTQ2 (aTP-mTQ2) expressed under the middle layer-specific *pGSO2* in the *rbohe rbohc* double mutant in anthers. PMC = pollen mother cell stage of pollen development. Scale bars: 10 µm.

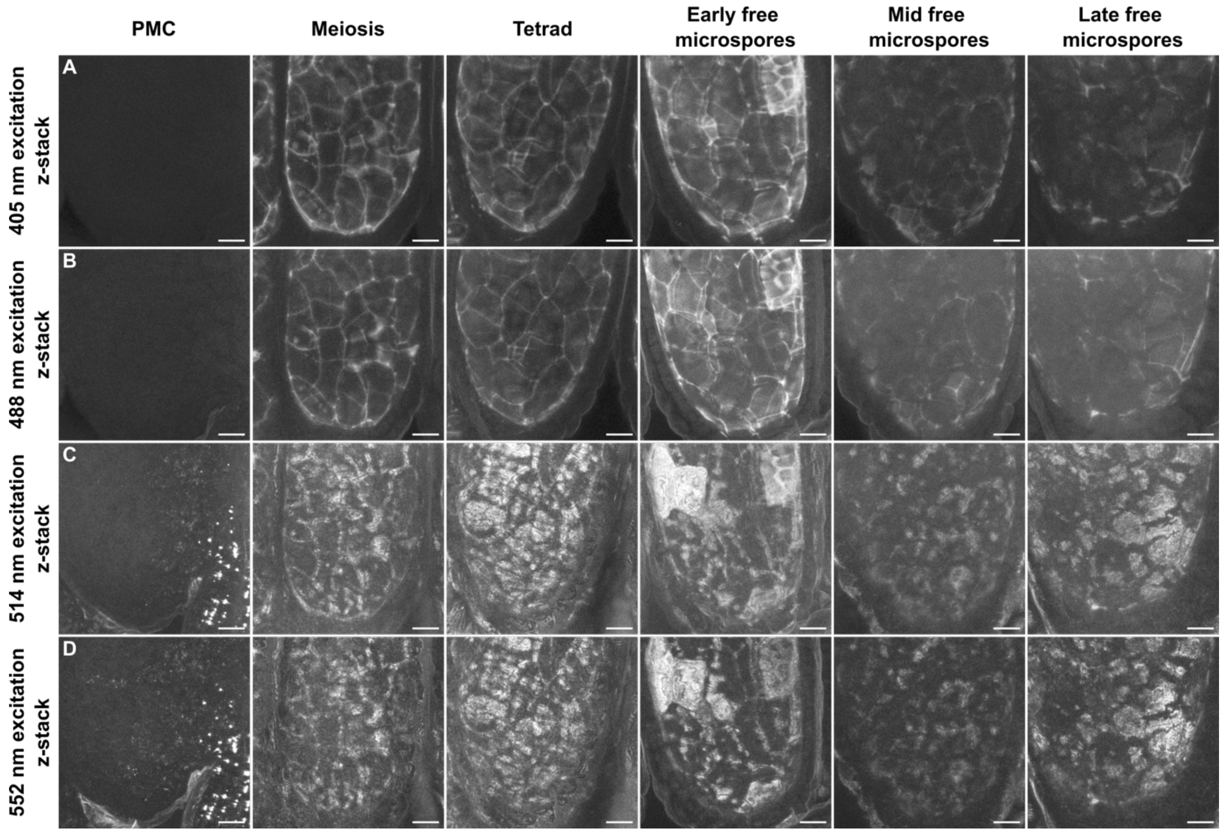

Fig. S3. **Peritapetal strip in *rbohe rbohc* double mutant anthers.** The PTS was exited at 405 nm (A), 488 nm (B), 514 nm (C) and 552 nm (D). Scale bars: 10 µm.

Development • Supplementary information

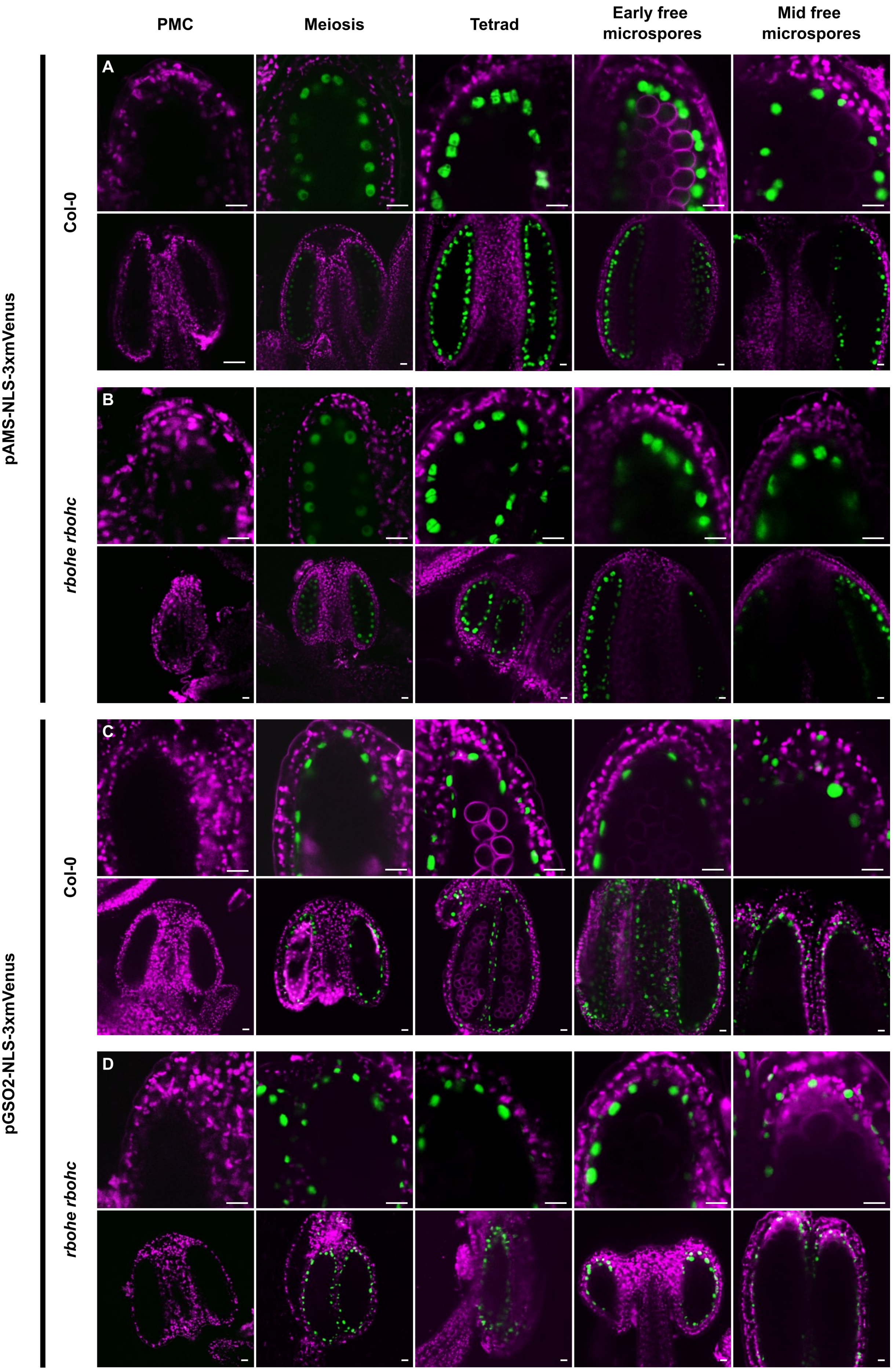

Development • Supplementary information

Fig. S4. **The expression of the *AMS* and *GSO2* promoters is identical in wild-type and *rbohe rbohc* double mutant anthers.** Expression of the *AMS* (A, C) and *GSO2* (B, D) promoters driving nuclear localized reporters in Col-0 (wild-type) (A, B) and *rbohe rbohc* double mutant (C, D) anthers. PMC = pollen mother cell stage of pollen development. Scale bars: 10 µm.

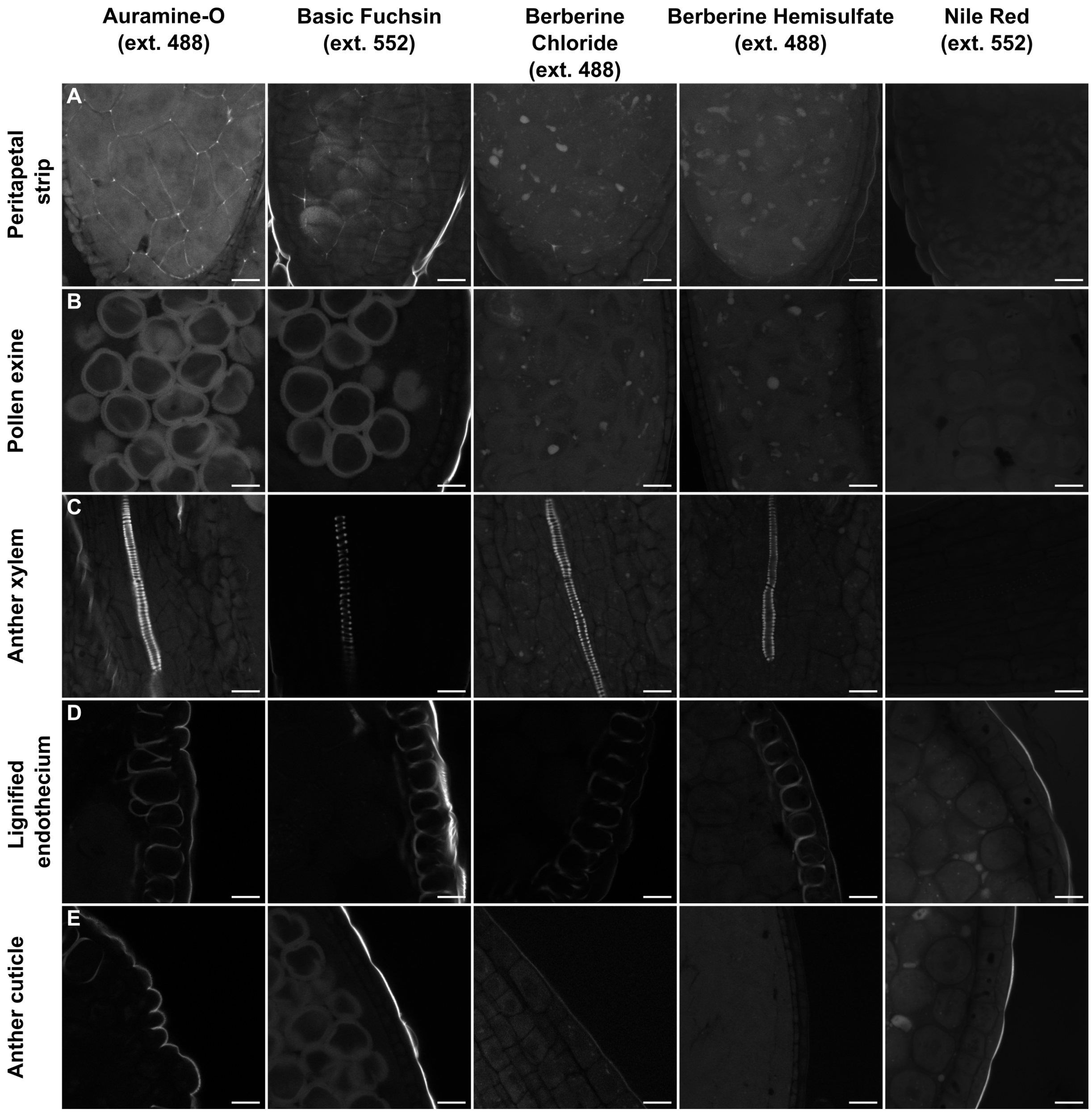

Fig. S5. Auramine-O, Basic Fuchsin, Berberine Chloride, Berberine Hemisulfate and Nile Red staining of the anthers. (A) Peritapetal strip at the early free microspore stage, (B) Pollen exine, (C) Anther xylem, (D) Lignified endothecium, (E) Anther cuticle. Scale bars: 10 µm.

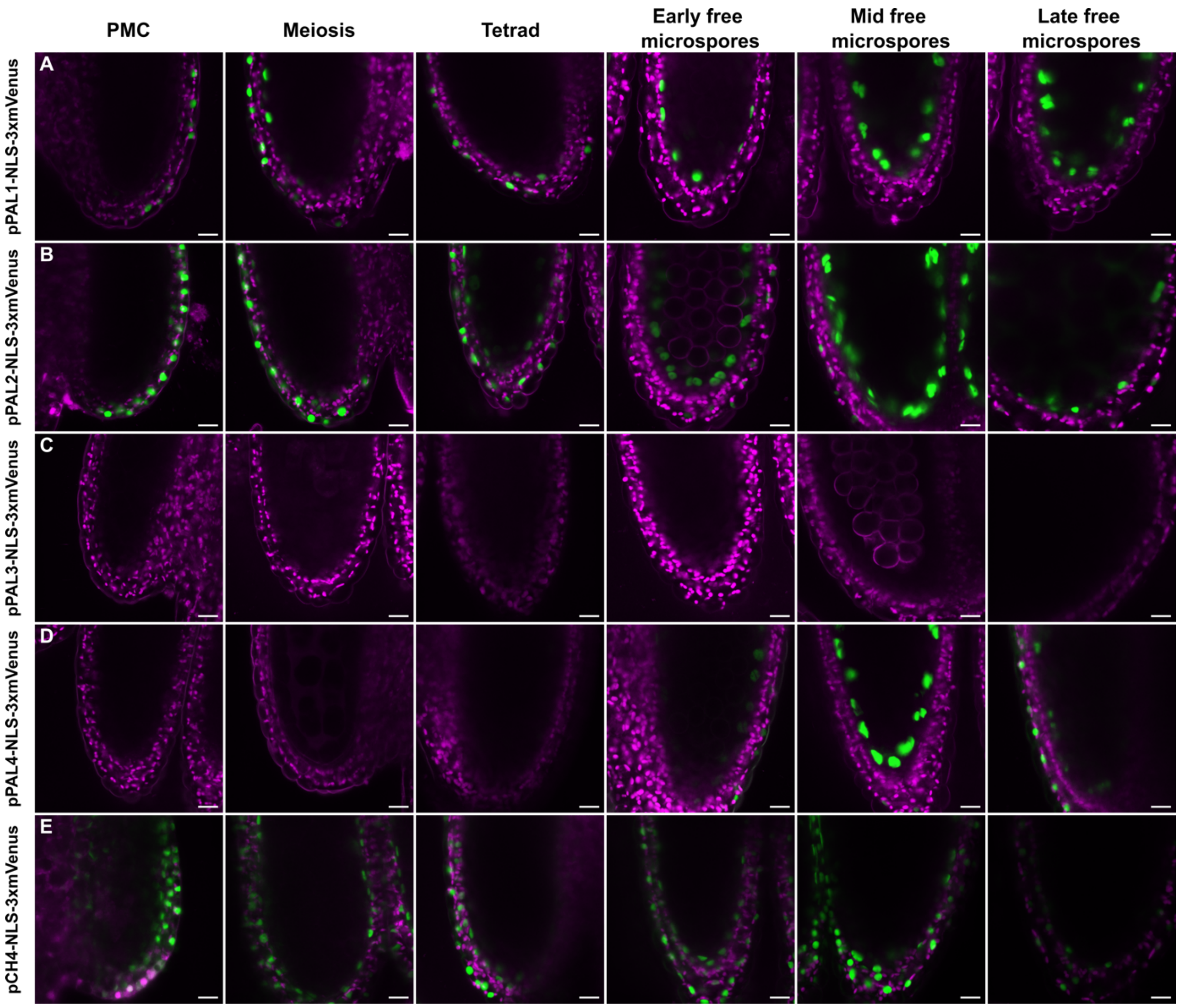

Fig. S6. Expression of the genes encoding components of the phenylpropanoid biosynthesis pathway in developing anthers. (A) *PAL1* expression with *pPAL1-NLS-3xmVenus* construct, (B) *PAL2* expression with *pPAL2-NLS-3xmVenus* construct, (C) *PAL3* expression with *pPAL3-NLS-3xmVenus* construct, (D) *PAL4* expression with *pPAL4-NLS- 3xmVenus* construct, (E) *C4H* expression with *pC4H-NLS-3xmVenus* construct. Scale bars: 10 µm.

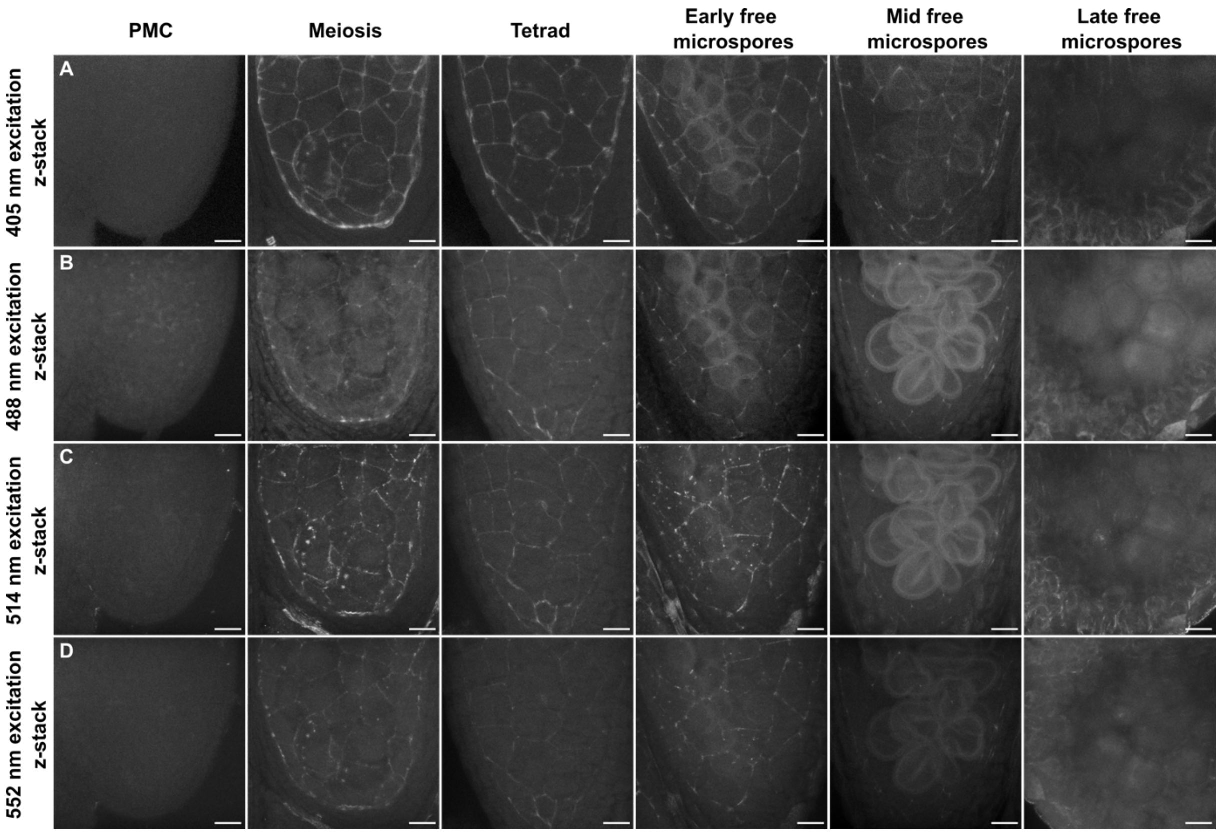

Fig. S7. Peritapetal strip in Ler wild-type anthers. The PTS was exited at 405 nm (A), 488 nm (B), 514 nm (C) and 552 nm (D). Scale bars: 10 µm.

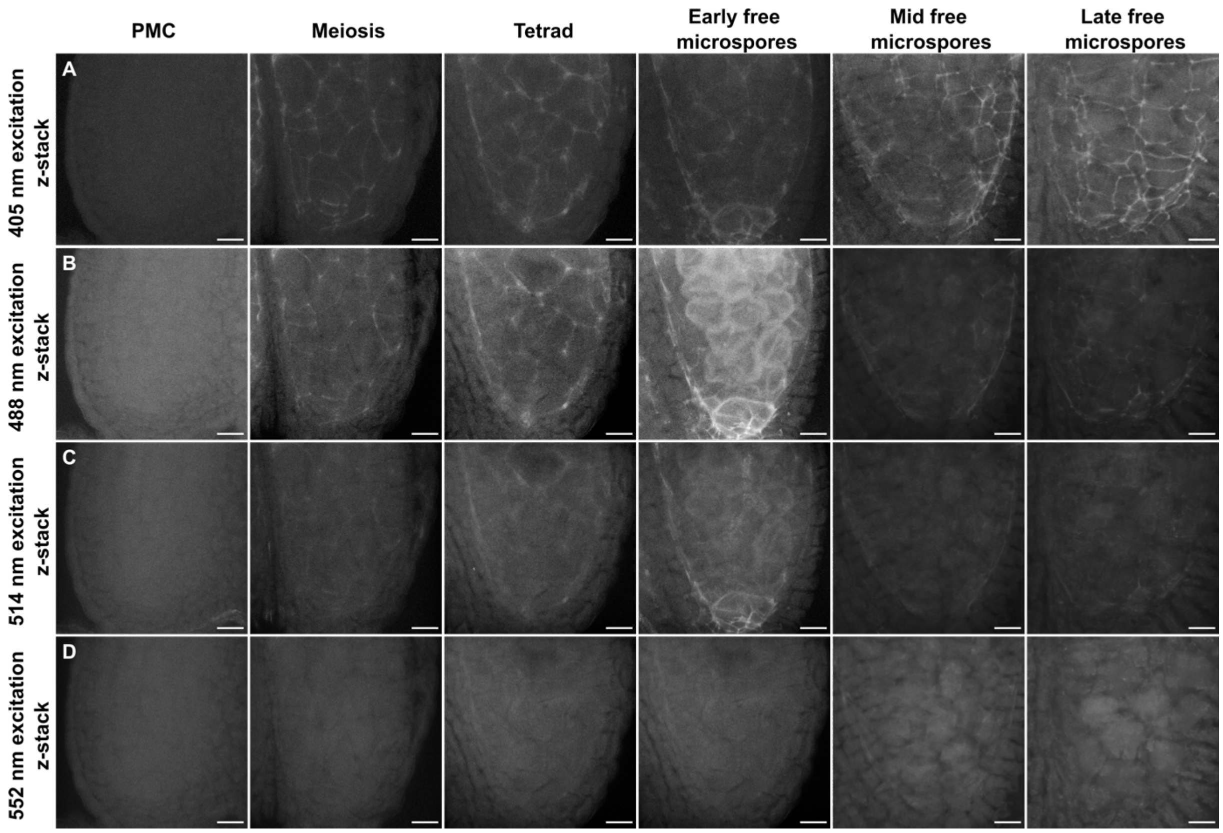

Fig. S8. Peritapetal strip in *ref3-2* mutant anthers. The PTS was exited at 405 nm (A), 488 nm (B), 514 nm (C) and 552 nm (D). Scale bars: 10 µm.

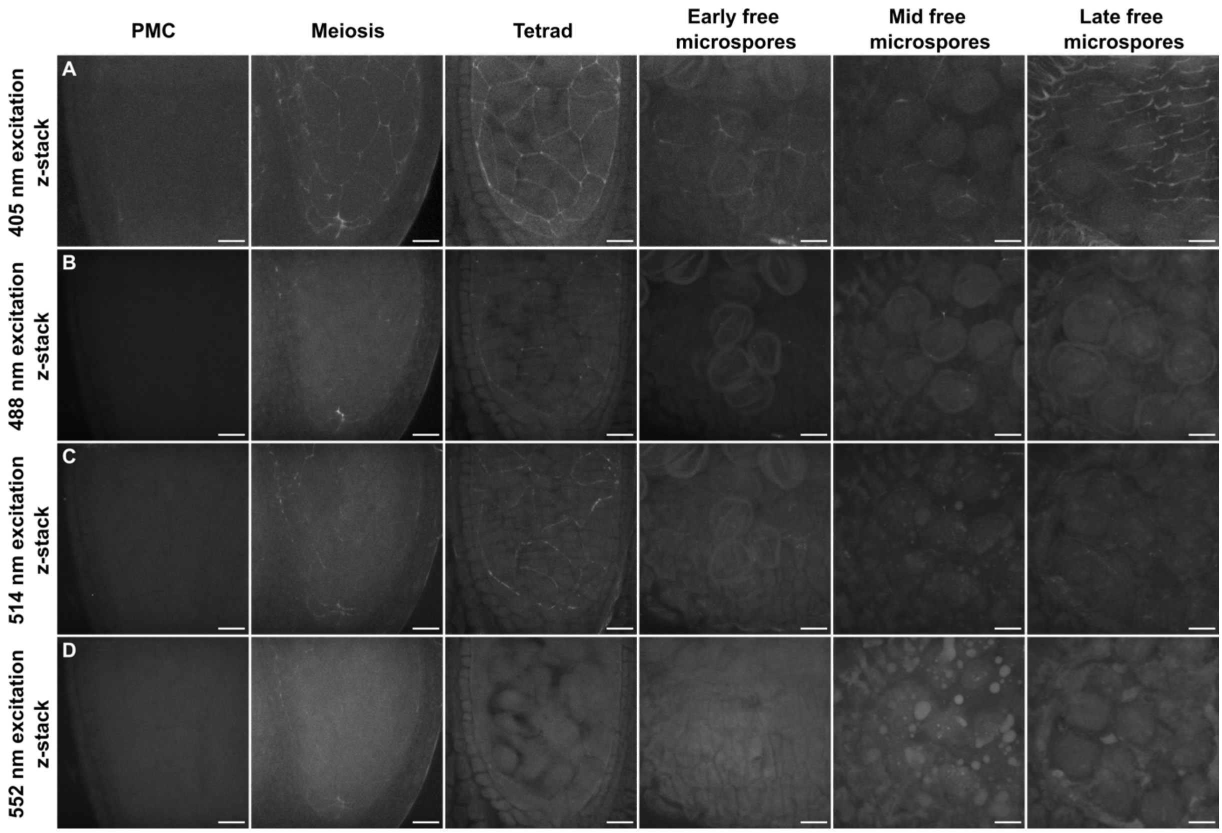

Fig. S9. Peritapetal strip in *ref3-1* mutant anthers. The PTS was exited at 405 nm (A), 488 nm (B), 514 nm (C) and 552 nm (D). Scale bars: 10 µm.

Development • Supplementary information

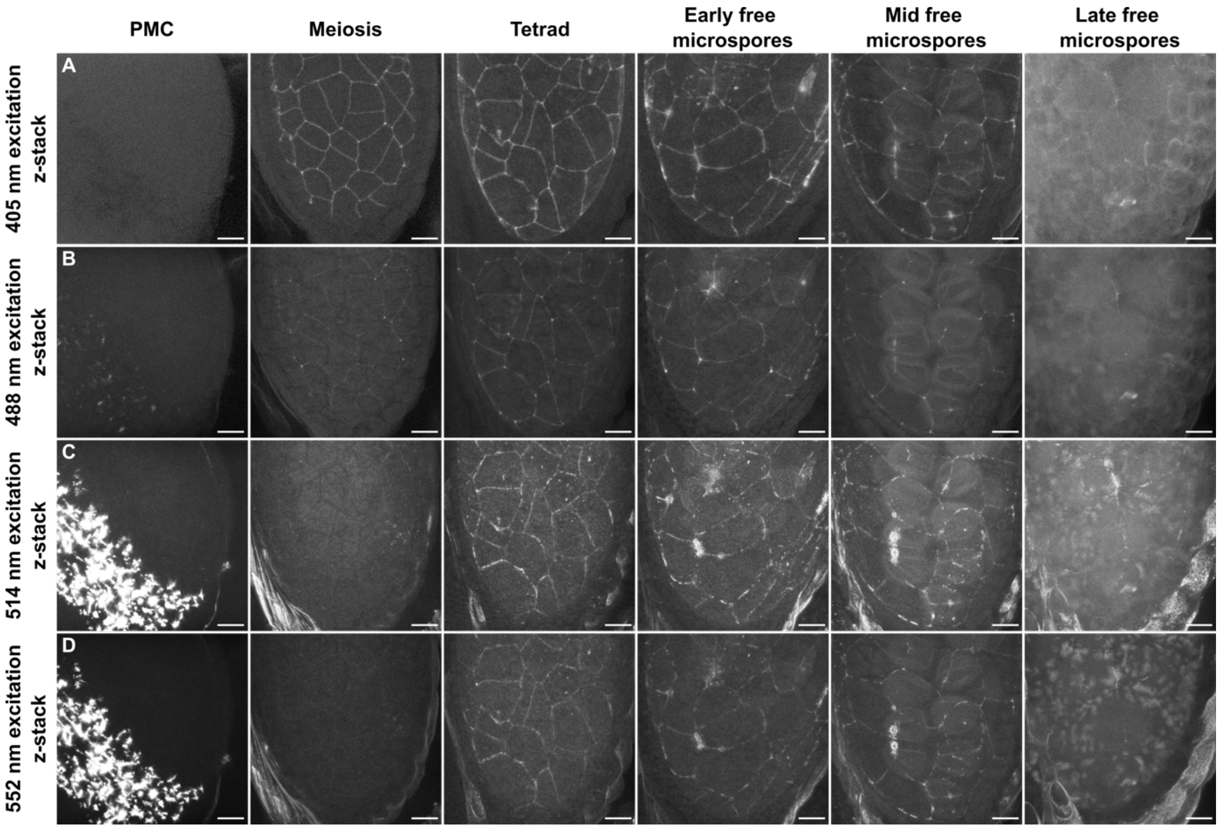

Fig. S10. Peritapetal strip in *ccr1* mutant anthers. The PTS was exited at 405 nm (A), 488 nm (B), 514 nm (C) and 552 nm (D). Scale bars: 10 µm.

Development • Supplementary information

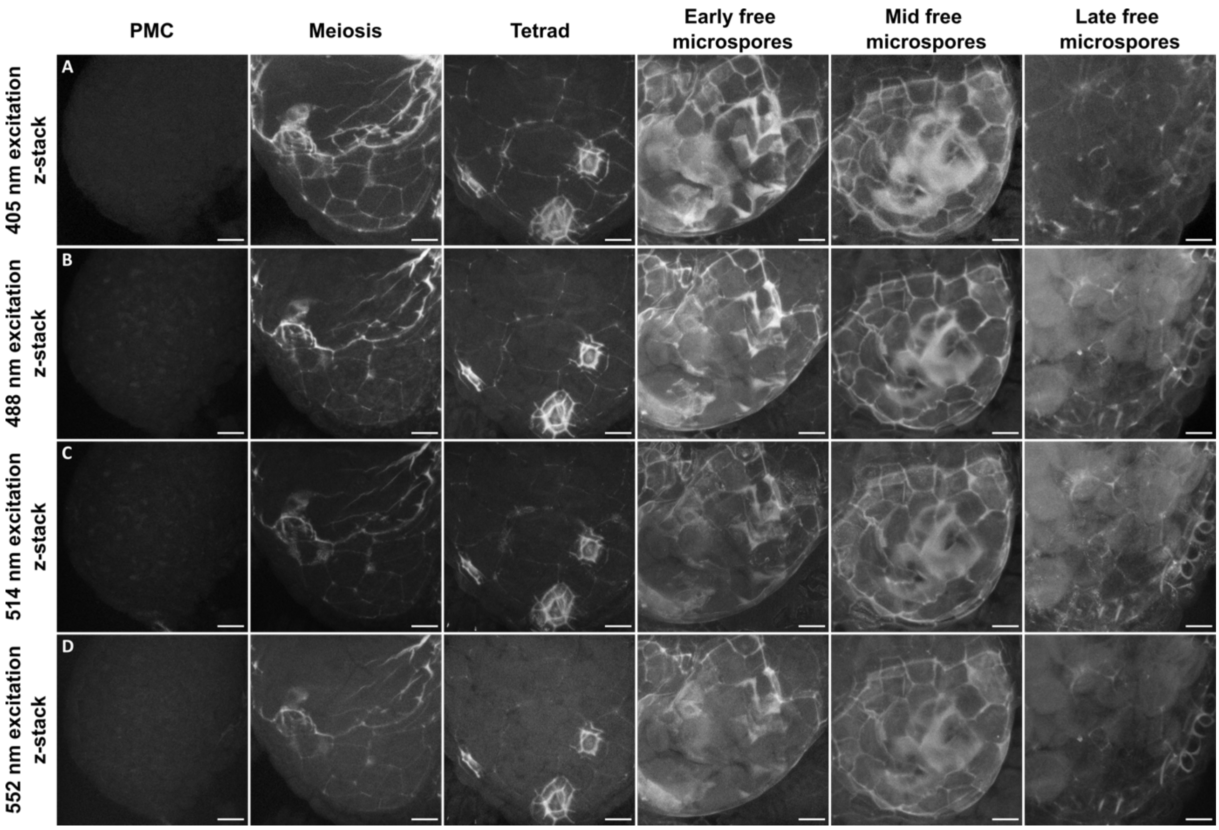

Fig. S11. Peritapetal strip in *rpk2-1* mutant anthers. The PTS was exited at 405 nm (A), 488 nm (B), 514 nm (C) and 552 nm (D). Scale bars: 10 µm.

Development • Supplementary information

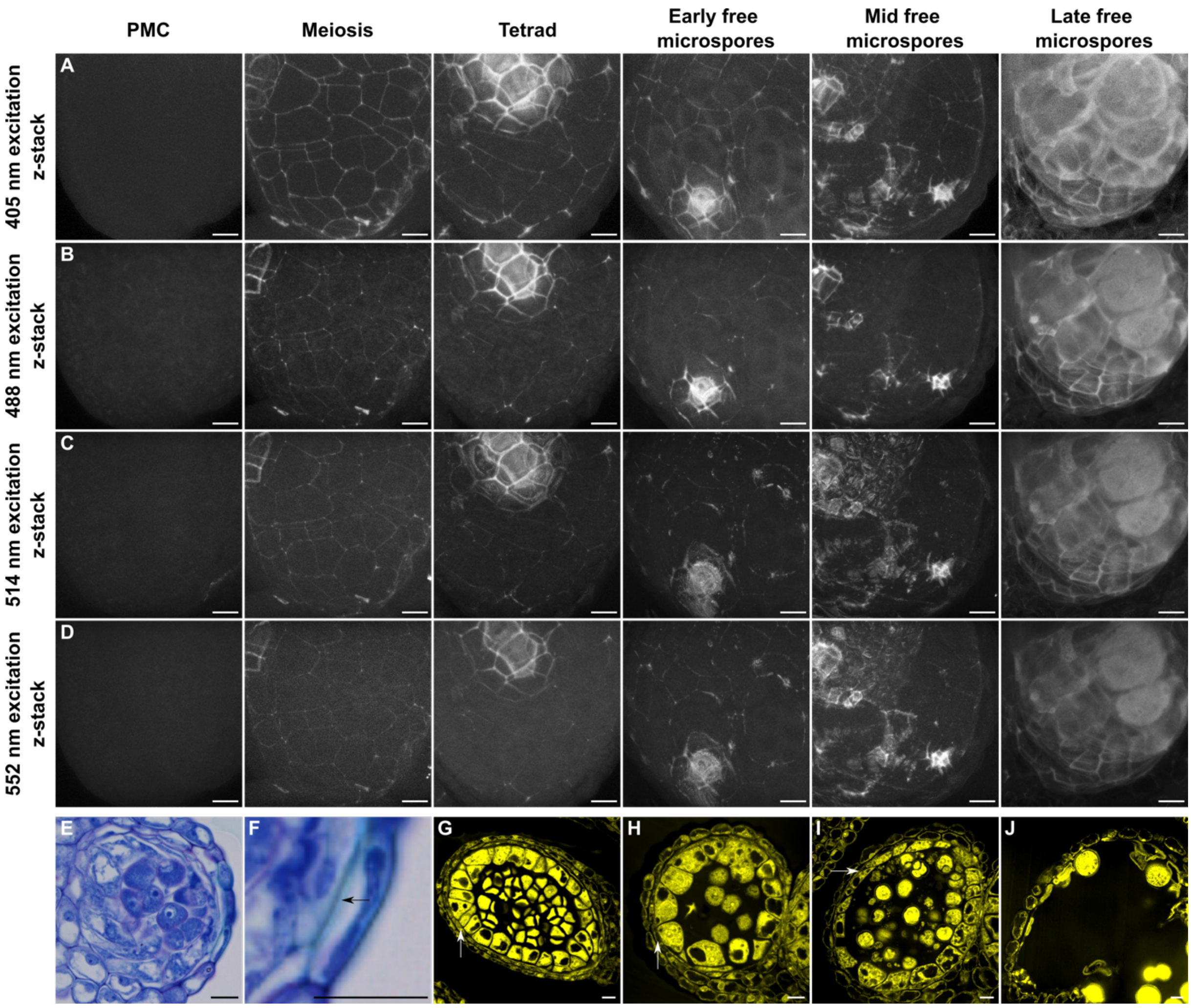

Fig. S12. Peritapetal strip in the *rpk2-2* mutant anthers. The PTS was exited at 405 nm (A), 488 nm (B), 514 nm (C) and 552 nm (D). (E-F) Ectopic lignin-like deposition around the middle layer in the *rpk2-2* mutant (arrow) visualized using Toluidine blue staining. Pollen and anther development in the *rpk2-2* mutant. Arrows indicate the single "hybrid" cell layer which replaces the endothecium and the middle layer in this mutant. Scale bars: 10 µm.

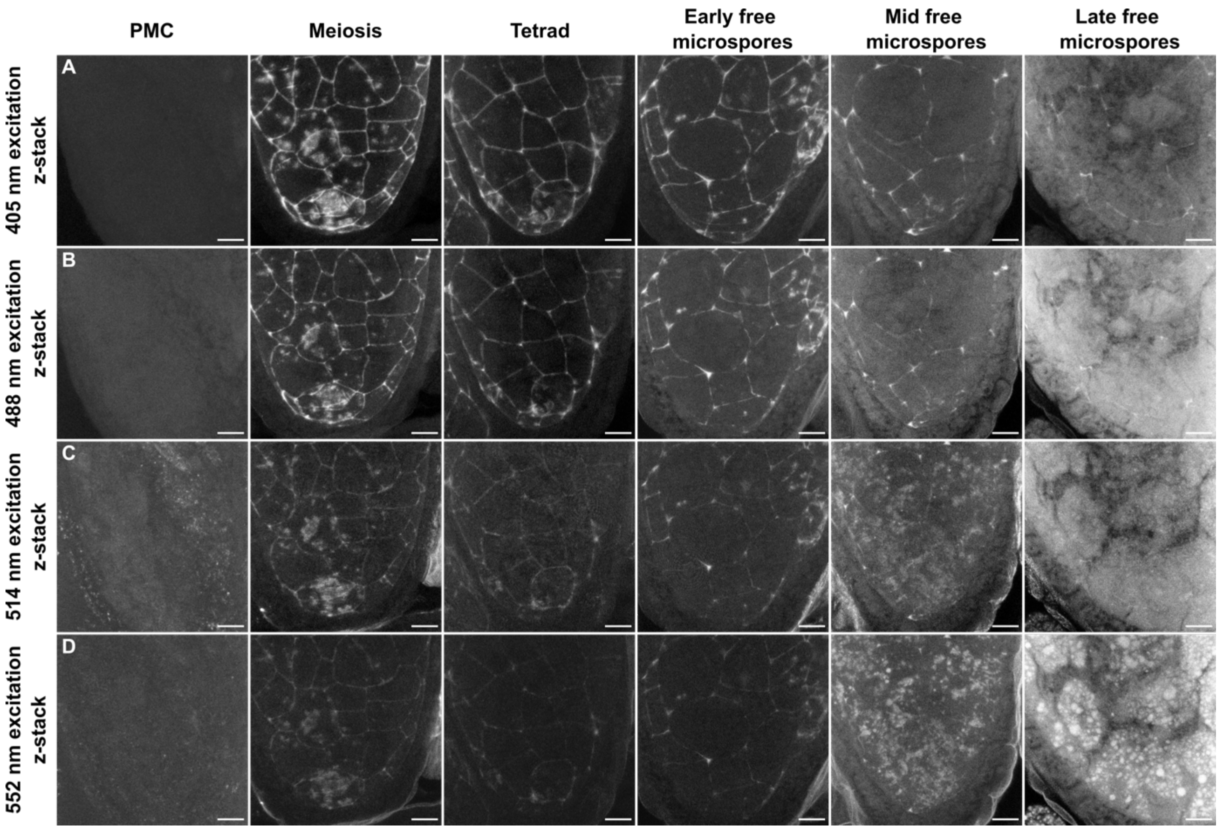

Fig. S13. Peritapetal strip in *ms2* mutant anthers. The PTS was exited at 405 nm (A), 488 nm (B), 514 nm (C) and 552 nm (D). Scale bars: 10 µm.

Development • Supplementary information

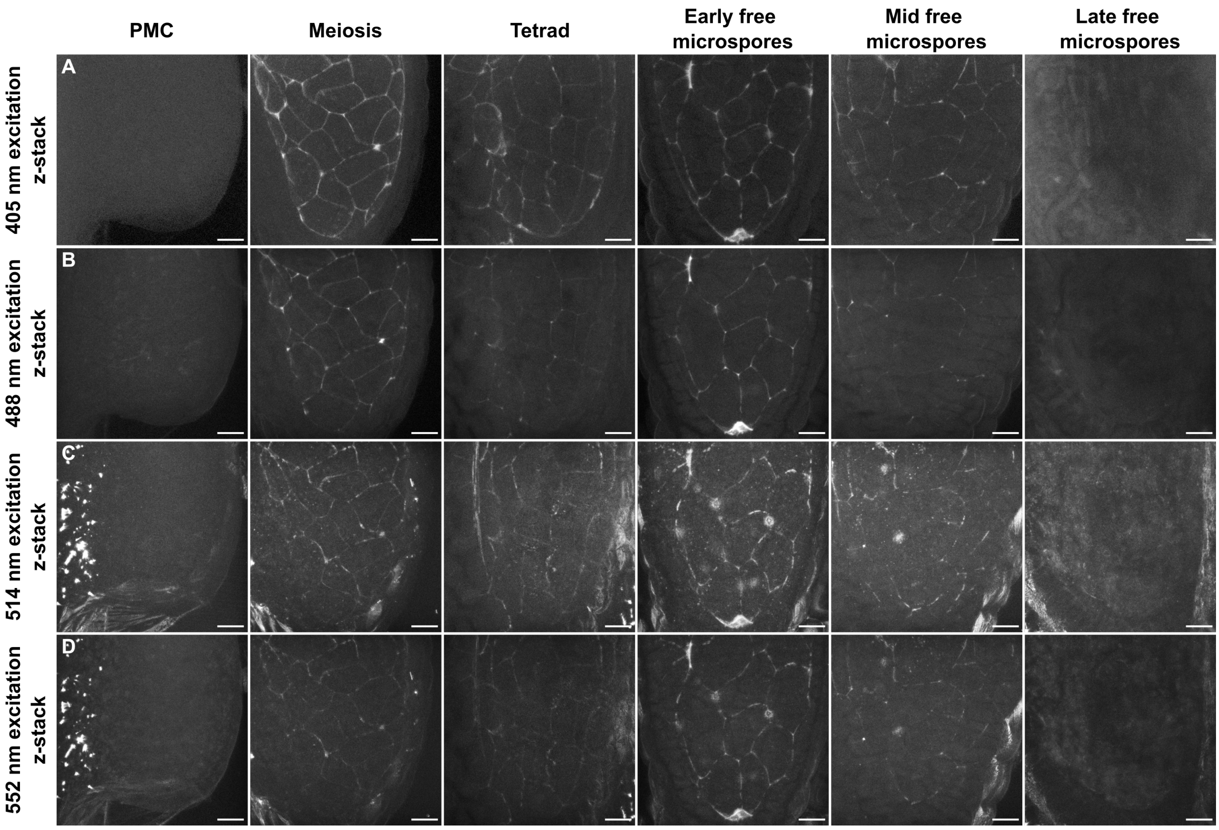

Fig. S14. Peritapetal strip in *acos5* mutant anthers. The PTS was exited at 405 nm (A), 488 nm (B), 514 nm (C) and 552 nm (D). Scale bars: 10 µm.

Development • Supplementary information

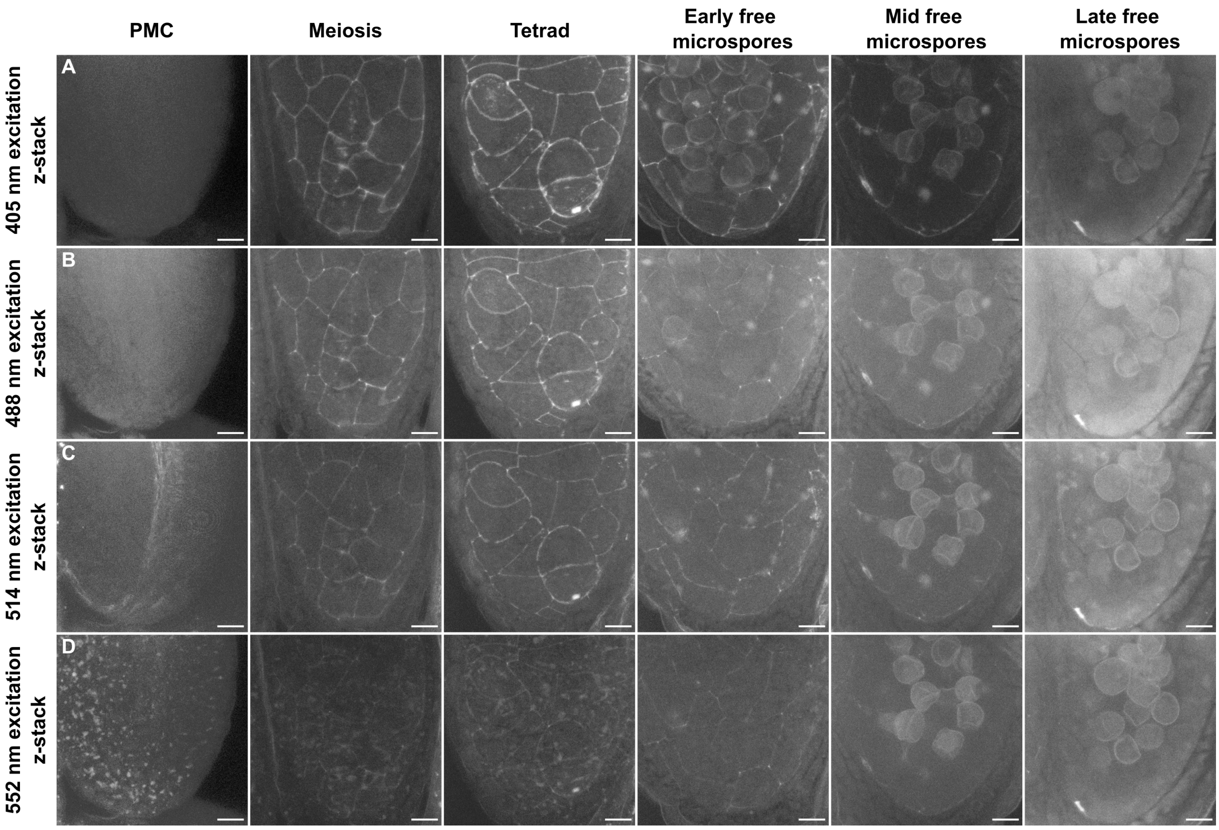

Fig. S15. Peritapetal strip in *dex2* mutant anthers. The PTS was exited at 405 nm (A), 488 nm (B), 514 nm (C) and 552 nm (D). Scale bars: 10 µm.

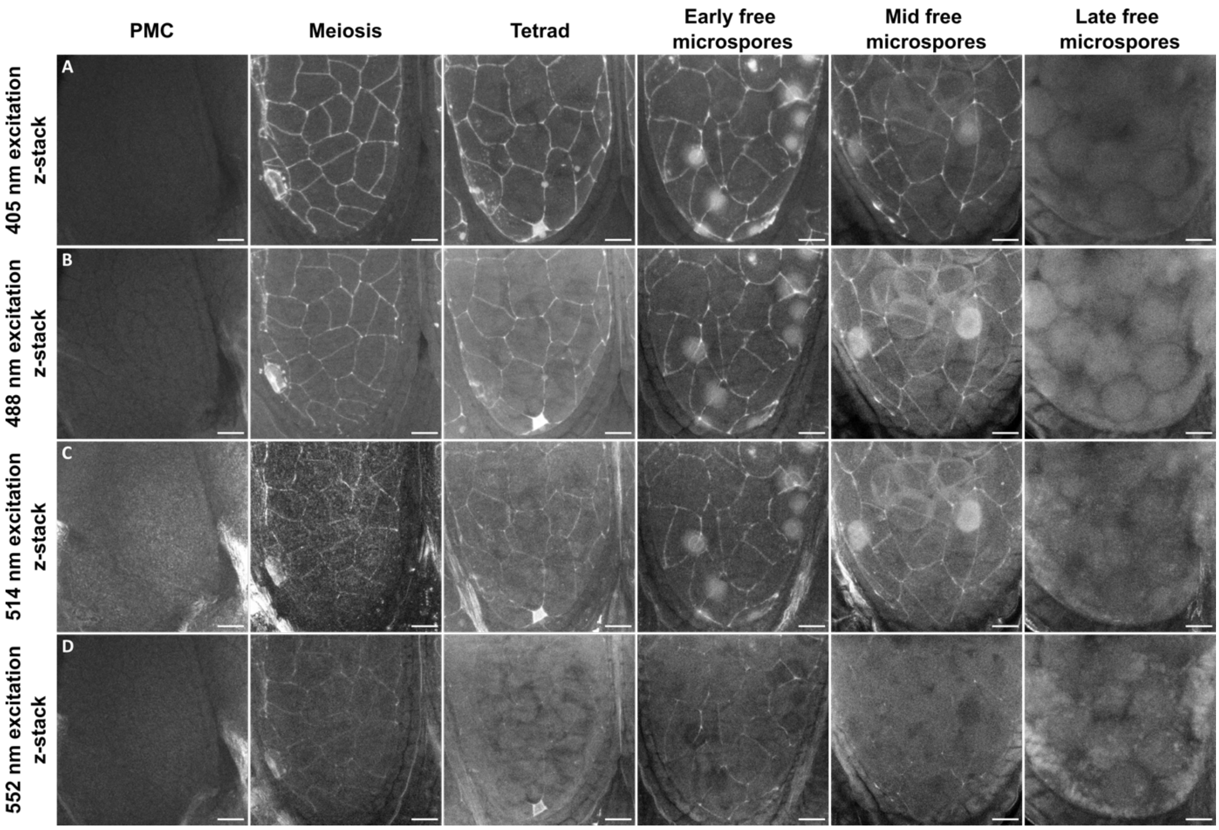

Fig. S16. Peritapetal strip in *pksb* mutant anthers. The PTS was exited at 405 nm (A), 488 nm (B), 514 nm (C) and 552 nm (D). Scale bars: 10 µm.

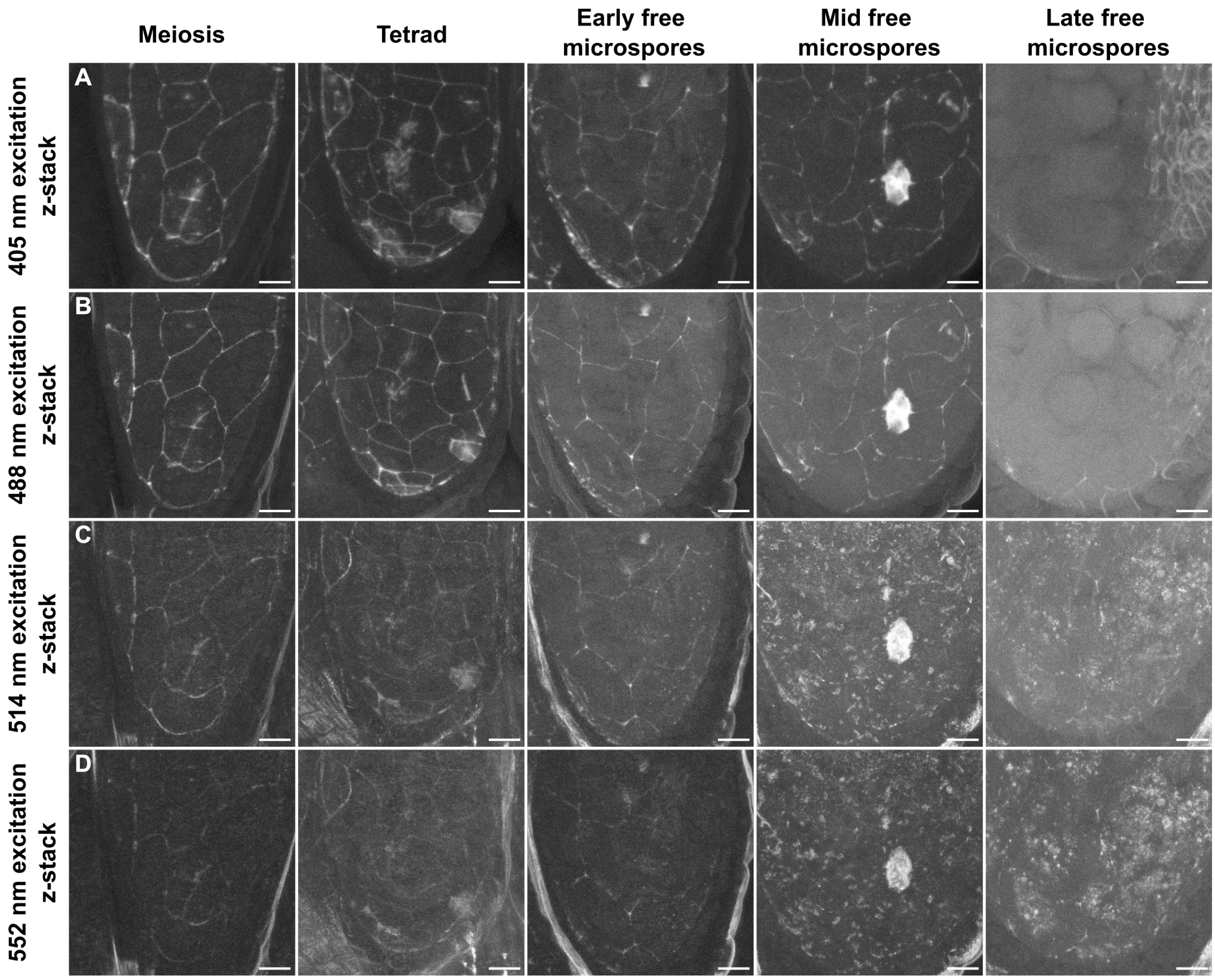

Fig. S17. Peritapetal strip in *tkpr2* mutant anthers. The PTS was exited at 405 nm (A), 488 nm (B), 514 nm (C) and 552 nm (D). Scale bars: 10 µm.

Table S1. Primer list

	Forward primer	Reverse primer	T-DNA primer/other info
**Genotyping primers**			
rbohe (SALK_150096)	GAGTTCGAGTTTCAGTCACGC	ATGCAAGTTGATGAGACCTCG	ATTTTGCCGATTTCGGAAC
rbohc (*rhd2-1*)	GAACATACATCTAATAAGTGTTCATCATGATGCACTGCA	GCAACATGTCGGCTCTAAGCAGACCAC	wild-type fragment but not the mutant can be digested by PstI into 40- and 200-bp fragments.
rpk2-1 (SALK_062412)	CAACGAAGAGCAATTTCCAAG	GATCTTTTGCTCGTCAATTGC	ATTTTGCCGATTTCGGAAC
rpk2-2 (SALK_039514)	GCCTTCTCGGTAAAGAAGGAG	GTAGGAATCTGGGGAATGGAG	ATTTTGCCGATTTCGGAAC
ccr1-3 (SALK_123689)	GTGTCGTAGAGGCTTTGCTTG	TTGTGGAAATATTTCCGGTTG	ATTTTGCCGATTTCGGAAC
acos5 (SK19167)	ATCATTTCAGACCTGACACCG	TTTCCGCAGTTTGTATCCATC	TTCTCATCTAAGCCCCCATTTGG
dex2-2 (SALK_119582)	CAAGGCTTCAGCATGTAAAGC	CATTCGGAACGTCTCTCGTAC	ATTTTGCCGATTTCGGAAC
pksb (GABI_454C04)	GCTTGGACTCAGTCCTGACAC	TAGGGAGATACGTGGGGAAAC	ATAATAACGCTGCGGACATCTACATTTT
tkpr2-1 (SALK_129453)	CAAATCACTTCTCGAACTCGG	GAAGGATCCAAATCCCAACTC	ATTTTGCCGATTTCGGAAC
**Cloning primers**			
pRBOHE	GGGGACAACTTTGTATAGAAAAGTTGGACGGCCTGCTAGCAAATGAT	GGGGACTGCTTTTTTGTACAAACTTGTCAGTGAGAGAGATTAACCAACC	
pTP-mTQ2 apoplastic signal fragment	GGGGACAAGTTTGTACAAAAAAGCAGGCTCAATGGGAGCTAACAAGTTGTTC	GCTCCTCGCCCTTGCTCACGGGAGCGTTAGTAAGAAGG	
pTP-mTQ2 mTQ2 sequence fragment	CCTTCTTACTAACGCTCCCGTGAGCAAGGGCGAGGAGC	GGGGACCACTTTGTACAAGAAAGCTGGGTGCTACTTGTACAGCTCGTCCAT	
mTQ2 cytoplasmic	GGGGACAAGTTTGTACAAAAAAGCAGGCTCAATGGTGAGCAAGGGCGAG	GGGGACCACTTTGTACAAGAAAGCTGGGTGCTACTTGTACAGCTCGTCCAT	

Development • Supplementary information
