# Supplementary material for: Anther development in Arabidopsis thaliana involves symplastic isolation and apoplastic gating of the tapetum-middle layer interface
Source: Development. 2022 Nov 16;149(22):dev200596. doi: 10.1242/dev.200596 (PMC10114112; doi:10.1242/dev.200596)
Supplement: Supplementary file 1 [file develop-149-200596_review_history.pdf]

# Anther development in *Arabidopsis thaliana* involves symplastic isolation and apoplastic gating of the tapetum-middle layer interface

Jekaterina Truskina, Sophy Boeuf, Joan Renard, Tonni Grube Andersen, Niko Geldner and Gwyneth Ingram
DOI: 10.1242/dev.200596

**Editor**: Yka Helariutta

**Review timeline**

| | |
|---|---|
| Original submission: | 3 February 2022 |
| Editorial decision: | 8 March 2022 |
| First revision received: | 10 August 2022 |
| Editorial decision: | 3 October 2022 |
| Second revision received: | 11 October 2022 |
| Accepted: | 24 October 2022 |


---

**Original submission**

First decision letter

MS ID#: DEVELOP/2022/200596

MS TITLE: Anther development in Arabidopsis thaliana involves symplastic isolation and apoplastic gating of the tapetum-middle layer interface.

AUTHORS: Jekaterina Truskina, Sophy Boeuf, Tonni Grube Andersen, Niko Geldner, and Gwyneth Ingram

I have now received all the referees' reports on the above manuscript, and have reached a decision. The referees' comments are appended below, or you can access them online: please go to BenchPress and click on the 'Manuscripts with Decisions' queue in the Author Area.

As you will see, the referees express considerable interest in your work, but have some significant criticisms and recommend a substantial revision of your manuscript before we can consider publication. If you are able to revise the manuscript along the lines suggested, which may involve further experiments, I will be happy receive a revised version of the manuscript. Your revised paper will be re-reviewed by one or more of the original referees, and acceptance of your manuscript will depend on your addressing satisfactorily the reviewers' major concerns. Please also note that Development will normally permit only one round of major revision.

We are aware that you may be experiencing disruption to the normal running of your lab that make experimental revisions challenging. If it would be helpful, we encourage you to contact us to discuss your revision in greater detail. Please send us a point-by-point response indicating where you are able to address concerns raised (either experimentally or by changes to the text) and where you will not be able to do so within the normal timeframe of a revision. We will then provide further guidance. Please also note that we are happy to extend revision timeframes as necessary.

Please attend to all of the reviewers' comments and ensure that you clearly highlight all changes made in the revised manuscript. Please avoid using 'Tracked changes' in Word files as these are lost

© 2022. Published by The Company of Biologists under the terms of the Creative Commons Attribution License (https://creativecommons.org/licenses/by/4.0/).

in PDF conversion. I should be grateful if you would also provide a point-by-point response detailing how you have dealt with the points raised by the reviewers in the 'Response to Reviewers' box. If you do not agree with any of their criticisms or suggestions please explain clearly why this is so.

Reviewer 1

*Advance summary and potential significance to field*

The authors present characterisation of the peritapetal membrane (PTM) via novel imaging techniques suggesting that the PTM is actually a cell wall structure that prevents apoplastic diffusion between the tapetum and middle cell layer of the developing anther. They establish that formation and the composition of the PTM is dependent on NADPH oxidases and cinnamate 4-hydroxylase, as well as components invovled with sporopollenin synthesis, and suggest that its appearance is correlated with symplastic and apoplastic isolation of the tapetal layer. In general, I think the manuscript identifies an interesting and critical feature of anther development, and that the different fluorescence profiles of the PTM in different mutations is a simple but powerful technique. However several key conclusions of the study rely on uncontrolled (or poorly controlled) experiments and others on correlation and description that would benefit from further quantification. Thus, while I think that the findings are very interesting, I think publication is premature.

*Comments for the author*

- Controls for experiments that describe symplastic/apoplastic isolation of the tapetum and middle layer:
i) The authors rely on the labelling of plasmodesmata with GFP-MCTP4 produced from the native promoter and characterise that labelling disappears between the middle layer and tapetum from the tetrad stage. While the conclusion is simply that this ‘could’ indicate symplastic isolation, little can be drawn from this observation given that the expression pattern of MCTP4 during anther development is not determined. Depending on the turnover rate of MCTPs in plasmodesmata, the same result could simply be due to down-regulation of expression of MCTP4, or given how little we understand about MCTPs that plasmodesmata in different cell types at different stages don’t recruit MCTP4.

ii) With respect to the experiments in which symplastic and apoplastic mTQ2 is driven from cell specific promoters and its mobility tracked, there are no expression controls. These experiments would require demonstrations that these promoters behaves as stated, i.e., the source of the ‘mobile’ proteins needs to be clearly defined. I sought this information in the preprint referred to but could not find any information relating to the AMS and GSO2 promoters. For tracking of protein mobility, this information is critical. Ideally this would be done by expression of a cell restricted protein simultaneously as the cell-to-cell mobile protein. However, when this is not possible, the expression profile should be determined in some way to allow comparison. Thus, these experiments are essentially uncontrolled within this manuscript. This point is particularly critical when similar experiments are performed with the rbohe,c double mutant as there is no demonstration that the promoter expression profiles are similar in the mutant context. Given the surprising observation that apoplastic mTQ2 can move from the tapetal layer outwards, but not from the middle layer inwards could be explained by a change in promoter expression patterns in the mutant background.
iii) The authors conclude that the tapetum is symplastically isolated from meiosis onwards. This is splitting hairs somewhat, but there is no expression of cytosolic mTQ2 from pAMS before meiosis so it is impossible to address this question before meiosis.

- The concept of gating and a barrier:
i) The authors comment in the discussion that the PTM is permeable to peptides and the title refers to the gating of the tapetum-middle layer interface. However, the work in this manuscript addresses the moment of fluorescent proteins and considers a binary situation – movement is possible or not and the PTM is described as a barrier. If the authors other work suggests the barrier is size dependent, this could be probed with dyes to track the permeability of the PTM. While I can see that this would be very difficult from the tapetal layer outwards, it seems possible to load a

© 2022. Published by The Company of Biologists under the terms of the Creative Commons Attribution License (https://creativecommons.org/licenses/by/4.0/).

dye from the surface of an intact anther, or via the vasculature, and track both apoplastic and symplastic permeability to smaller molecules.

ii) The authors also state in the discussion that the PTM must allow diffusion of sugars and nutrients, but given that like the apoplastic pathway they have only tracked the symplastic path with a fluorescent protein. If the size limit argument is plausible for the apoplast, it must also be for the symplast and without experiments with smaller probes, this statement is unfounded.

- Description of PTM fluorescence with different excitations in different mutants:
I think the difference fluorescence from the different excitation wavelengths is very interesting and a strong indicator of a difference in PTM composition.
However, the experiments are very descriptive, with single images to support the conclusion. I would encourage the authors to consider a method of quantification for this, whether that be the intensity of different emissions within an image series, or relative to wild-type fluorescence. With the former strategy, the data having multiple measures per sample allows the data to be internally normalised.

Minor comments

- The authors maintain the PTM name for the boundary, but they clearly show it's not membrane? I would encourage them to propose new terminology.
- I would move the ref3-2, 3-1 analysis to the same section as the C4H expression analysis as it builds a strong piece of evidence for the involvement of C4H in the generation of PTM – these mutants seem to have the strongest PTM phenotype. I would make more of this.
- Fig 1K-O – labels on the cell features would aid interpretation (cytosol, cell wall, PM etc) especially between PMC and meiosis when the cell appearance changes dramatically

Reviewer 2

*Advance summary and potential significance to field*

The authors proved the symplastical isolation of the tapetum and middle layer in anthers and the existence of an apoplastic structure peritapetal membrane (PTM) between the two layers by various histological and microscopy imaging methods. Further they analyzed the components and function of the PTM, and identified potential key regulators required for PTM formation. It extends our knowledge of the communication control of tapetum and surrounding cell layers during pollen development.

*Comments for the author*

Major concerns:

1. In Fig.2, the authors aim to show the loss of plasmodesmata between the middle layer and tapetum using pAtMCTP4:GFP-AtMCTP4 as the plasmodesmata marker. So, the data showing the detailed expression pattern of AtMCTP4 gene in anther are required because the absence of MCTP4 does not mean the absence of plasmodesmata.
2. Similarly, the tissue activity of AMS promoter should be analyzed in rbohe rbohc double mutant to prove the ectopic signal of mTQ2 around endothecium and epidermis cells (Fig 4g) is not due to the change of AMS expression.
3. The authors also stated the contradictory result when studying the aTP-mTQ2 movement using AMS and GSO2 promoters respectively. Thus, I suggest providing one more analysis using another promoter.

Minor points:

1. The authors concluded the PTM may contain phenolic compounds by detecting the formation of PTM in various related mutants. In addition, the movement assay of fluorescent molecules in these mutants would be better to prove the functional defect. This could be just a suggestion if it will take a long time for this analysis.

© 2022. Published by The Company of Biologists under the terms of the Creative Commons Attribution License (https://creativecommons.org/licenses/by/4.0/).

Reviewer 3

*Advance summary and potential significance to field*

Truskina et al., report the interesting discover on the compartmentation of anthers by experimentally showing that Arabidopsis tapetum and the developing pollen are symplastically isolated from each other, and from other sporophytic tissues, from meiosis onwards. In addition, an apoplastic structure the peritapetal membrane (PTM), was structurally and compositionally characterised separating the central domain of the anther (tapetum and the pollen grains) from other maternal cell layers. Furthermore the authors proved that two NADPH oxidases, RBOHE and RBOHC, are required for the formation and barrier functions of the PTM. The pollen wall forming genes are needed to consolidate the structure of PTM. Overall this is an interesting work with well-designed experiments, providing the insight into how plant male organ anther forms structure for chemical delivery and signaling during pollen formation.

*Comments for the author*

Truskina et al., report the interesting discover on the compartmentation of anthers by experimentally showing that Arabidopsis tapetum and the developing pollen are symplastically isolated from each other, and from other sporophytic tissues, from meiosis onwards. In addition, an apoplastic structure the peritapetal membrane (PTM), was structurally and compositionally characterised separating the central domain of the anther (tapetum and the pollen grains) from other maternal cell layers. Furthermore the authors proved that two NADPH oxidases, RBOHE and RBOHC, are required for the formation and barrier functions of the PTM. The pollen wall forming genes are needed to consolidate the structure of PTM. Overall this is an interesting work with well-designed experiments providing the insight into how plant male organ anther forms structure for chemical delivery and signaling during pollen formation.

Comment: I am not fully convinced by the statement that the middle layer appears to be key player for the PTM formation by supplying specific phenolic compounds. I assume that tapetum may also has a role in synthesizing PTM as tapetum is a active tissue for the synthesis of phenolic compounds.

---

**First revision**

Author response to reviewers' comments

Reviewer 1 Advance Summary and Potential Significance to Field:
The authors present characterisation of the peritapetal membrane (PTM) via novel imaging techniques suggesting that the PTM is actually a cell wall structure that prevents apoplastic diffusion between the tapetum and middle cell layer of the developing anther. They establish that formation and the composition of the PTM is dependent on NADPH oxidases and cinnamate 4-hydroxylase, as well as components invovled with sporopollenin synthesis, and suggest that its appearance is correlated with symplastic and apoplastic isolation of the tapetal layer. In general, I think the manuscript identifies an interesting and critical feature of anther development, and that the different fluorescence profiles of the PTM in different mutations is a simple but powerful technique. However, several key conclusions of the study rely on uncontrolled (or poorly controlled) experiments and others on correlation and description that would benefit from further quantification. Thus, while I think that the findings are very interesting, I think publication is premature.

***-We would like to thank this reviewer for their thoughtful comments, which we have done our best to address below. In response to this reviewer's comments, and the comments of Reviewer 2, we have renamed the Peritapetal Membrane (PTM) the Peritapetal Strip (PTS).***

© 2022. Published by The Company of Biologists under the terms of the Creative Commons Attribution License (https://creativecommons.org/licenses/by/4.0/).

Reviewer 1 Comments for the Author:
- Controls for experiments that describe symplastic/apoplastic isolation of the tapetum and middle layer:

i) The authors rely on the labelling of plasmodesmata with GFP-MCTP4 produced from the native promoter and characterise that labelling disappears between the middle layer and tapetum from the tetrad stage. While the conclusion is simply that this 'could' indicate symplastic isolation, little can be drawn from this observation given that the expression pattern of MCTP4 during anther development is not determined. Depending on the turnover rate of MCTPs in plasmodesmata, the same result could simply be due to down-regulation of expression of MCTP4, or given how little we understand about MCTPs that plasmodesmata in different cell types at different stages don't recruit MCTP4.

***We appreciate this comment. In particular we agree with the fact that it is possible that certain cell types may not recruit MCTP4 to their plasmodesmata. Given the fact that tapetal cells are thought to lose their cell walls at around the tetrad stage (Quilichini et al., 2014) and that plasmodesmata between tapetal cells are massively remodeled from around this time to produce intracellular connections in several species, it is entirely possible that MCTP4 localisation would be uninformative even in the presence of expression of the MCTP4 gene. We feel that the direct measures of intercellular protein mobility that we show are more informative in this regard. For these reasons we have removed the MCTP4 localization data from the manuscript.***

ii) With respect to the experiments in which symplastic and apoplastic mTQ2 is driven from cell specific promoters and its mobility tracked, there are no expression controls. These experiments would require demonstrations that these promoters behaves as stated, i.e., the source of the 'mobile' proteins needs to be clearly defined. I sought this information in the preprint referred to but could not find any information relating to the AMS and GSO2 promoters.

***We apologize for this. Extended data showing the expression of the GSO2 and AMS promoters is presented in the supplemental figures of this paper, which has now been published . We have also included images in the new supplemental Figure S4.***
***Proc Natl Acad Sci U S A. 2022 May 31;119(22):e2201446119. doi: 10.1073/pnas.2201446119.[1]***

For tracking of protein mobility, this information is critical. Ideally this would be done by expression of a cell restricted protein simultaneously as the cell- to-cell mobile protein. However, when this is not possible, the expression profile should be determined in some way to allow comparison. Thus, these experiments are essentially uncontrolled within this manuscript. This point is particularly critical when similar experiments are performed with the rbohe,c double mutant as there is no demonstration that the promoter expression profiles are similar in the mutant context. Given the surprising observation that apoplastic mTQ2 can move from the tapetal layer outwards, but not from the middle layer inwards could be explained by a change in promoter expression patterns in the mutant background.

***The reviewer is absolutely correct. To address this comment (and the identical comment from reviewer 2), we have now transformed the GSO2 and AMS reporter constructs directly into the rbohe,c double mutant. The results are presented in new supplementary figure S4.***

iii) The authors conclude that the tapetum is symplastically isolated from meiosis onwards. This is splitting hairs somewhat, but there is no expression of cytosolic mTQ2 from pAMS before meiosis so it is impossible to address this question before meiosis.

***Our study addresses inter-layer communication post meiosis. We agree with the reviewers' comments, but we feel that addressing communication prior to mitosis, when the PTS is not present, falls out with the scope of our study.***

- The concept of gating and a barrier:
i) The authors comment in the discussion that the PTM is permeable to peptides and the title refers to the gating of the tapetum-middle layer interface. However, the work in this manuscript addresses the moment of fluorescent proteins and considers a binary situation – movement is

© 2022. Published by The Company of Biologists under the terms of the Creative Commons Attribution License (https://creativecommons.org/licenses/by/4.0/).

possible or not and the PTM is described as a barrier. If the authors other work suggests the barrier is size dependent, this could be probed with dyes to track the permeability of the PTM. While I can see that this would be very difficult from the tapetal layer outwards, it seems possible to load a dye from the surface of an intact anther, or via the vasculature, and track both apoplastic and symplastic permeability to smaller molecules.

***We fully agree with this comment, and indeed have endeavored to carry out this type of study several times. However, because the anther is covered with a cuticle, external loading has proved very complicated, whilst feeding experiments involving the filament have given extremely variable results, which are additionally complex to visualize in intact anthers. As a result, we feel that an in-depth quantitative analysis of the permeability properties of the PTS would likely require a level of technical optimization that would be very difficult for us to achieve in the current time-frame.***

ii) The authors also state in the discussion that the PTM must allow diffusion of sugars and nutrients, but given that like the apoplastic pathway, they have only tracked the symplastic path with a fluorescent protein. If the size limit argument is plausible for the apoplast, it must also be for the symplast and without experiments with smaller probes, this statement is unfounded.

***We have toned down this statement. However, we would like to point out that although the permeability properties of apoplastic "barriers" (where they exist) are largely determined by their biochemical composition and can be very variable depending upon the barrier, and the diffusing molecule, symplastic communication can be fully lost if symplastic connections (plasmodesmata) no longer exist. Several reports in the literature have suggested that plasmodesmata do not exist between tapetal cells and neighboring cell layers post meiosis in both Arabidopsis and other species [2, 3]. We have now cited these explicitly in our manuscript. Thus, although symplastic exchanges are undoubtedly limited by variable size exclusion limits where plasmodesmata do exist, symplastic barriers (sensu stricto) caused by plasmodesmatal loss, can also exist. We propose that this is likely the case between the tapetum and the middle layer.***

- Description of PTM fluorescence with different excitations in different mutants:
I think the difference fluorescence from the different excitation wavelengths is very interesting and a strong indicator of a difference in PTM composition.
However, the experiments are very descriptive, with single images to support the conclusion. I would encourage the authors to consider a method of quantification for this, whether that be the intensity of different emissions within an image series, or relative to wild-type fluorescence. With the former strategy, the data having multiple measures per sample allows the data to be internally normalised.

***We agree that it would be interesting to carry out this type of analysis. However, given the heterogeneity of the signal in some of the mutant backgrounds, and, indeed, within even the wild type PTS, we have been unable to generate a pipeline that allows a convincing quantification of these signals. Our qualitative data are, we feel, convincing, and our conclusions are based on multiple images from multiple anthers.***

Minor comments
- The authors maintain the PTM name for the boundary, but they clearly show it's not membrane? I would encourage them to propose new terminology.

***We agree with this reviewer, and propose to call the PTM the Peritapetal Strip (PTS). This will also avoid the use of the acronym PTM, which is commonly used to denote Post-Transcriptional Modifications.***

- I would move the ref3-2, 3-1 analysis to the same section as the C4H expression analysis as it builds a strong piece of evidence for the involvement of C4H in the generation of PTM – these mutants seem to have the strongest PTM phenotype. I would make more of this.

***We agree with this comment and have changed the text and figures accordingly.***

© 2022. Published by The Company of Biologists under the terms of the Creative Commons Attribution License (https://creativecommons.org/licenses/by/4.0/).

- Fig 1K-O – labels on the cell features would aid interpretation (cytosol, cell wall, PM etc) especially between PMC and meiosis when the cell appearance changes dramatically

***We have made the requested changes.***

Reviewer 2 Advance Summary and Potential Significance to Field:
The authors proved the symplastical isolation of the tapetum and middle layer in anthers and the existence of an apoplastic structure peritapetal membrane (PTM) between the two layers by various histological and microscopy imaging methods. Further they analyzed the components and function of the PTM, and identified potential key regulators required for PTM formation. It extends our knowledge of the communication control of tapetum and surrounding cell layers during pollen development.

***We would like to thank this reviewer for their positive assessment of our work.***

Reviewer 2 Comments for the Author:
Major concerns:
1. In Fig.2, the authors aim to show the loss of plasmodesmata between the middle layer and tapetum using pAtMCTP4:GFP-AtMCTP4 as the plasmodesmata marker. So, the data showing the detailed expression pattern of AtMCTP4 gene in anther are required because the absence of MCTP4 does not mean the absence of plasmodesmata.

***This comment reflects almost exactly the first comment of reviewer 1. For the reasons evoked in our response to reviewer 1, we agree with the reviewer, and we have removed this data from the manuscript.***

2. Similarly, the tissue activity of AMS promoter should be analyzed in rbohe rbohc double mutant to prove the ectopic signal of mTQ2 around endothecium and epidermis cells (Fig 4g) is not due to the change of AMS expression.

***Again, this issue was raised by reviewer 1 and has been addressed in our revised manuscript by introducing appropriate expression markers into the rbohe rbohc double mutant (see figure S4).***

3. The authors also stated the contradictory result when studying the aTP-mTQ2 movement using AMS and GSO2 promoters respectively. Thus, I suggest providing one more analysis using another promoter.

***We are aware of no other promoter that could be used to replace that of GSO2 other than the promoter of GSO1, which is more weakly expressed. Indeed, since published the expression profile for GSO2 we have received numerous requests for our constructs from people interested in having a middle layer- expressed marker. We feel that our explanation for this phenomenon is plausible.***

Minor points:
1. The authors concluded the PTM may contain phenolic compounds by detecting the formation of PTM in various related mutants. In addition, the movement assay of fluorescent molecules in these mutants would be better to prove the functional defect. This could be just a suggestion if it will take a long time for this analysis.

***Again, this point was raised by reviewer 1. However we agree with reviewer 2 that including these experiments as future perspectives would be useful and have changed the discussion section accordingly***

Reviewer 3 Advance Summary and Potential Significance to Field:
Truskina et al., report the interesting discover on the compartmentation of anthers by experimentally showing that Arabidopsis tapetum and the developing pollen are symplastically isolated from each other, and from other sporophytic tissues, from meiosis onwards. In addition, an apoplastic structure the peritapetal membrane (PTM), was structurally and compositionally characterised separating the central domain of the anther (tapetum and the pollen grains) from

© 2022. Published by The Company of Biologists under the terms of the Creative Commons Attribution License (https://creativecommons.org/licenses/by/4.0/). 

other maternal cell layers. Furthermore the authors proved that two NADPH oxidases, RBOHE and RBOHC, are required for the formation and barrier functions of the PTM. The pollen wall forming genes are needed to consolidate the structure of PTM. Overall this is an interesting work with well-designed experiments, providing the insight into how plant male organ anther forms structure for chemical delivery and signaling during pollen formation.

***We would like to thank this reviewer for their positive assessment of our work.***

Reviewer 3 Comments for the Author:
Truskina et al., report the interesting discover on the compartmentation of anthers by experimentally showing that Arabidopsis tapetum and the developing pollen are symplastically isolated from each other, and from other sporophytic tissues, from meiosis onwards. In addition, an apoplastic structure the peritapetal membrane (PTM), was structurally and compositionally characterised separating the central domain of the anther (tapetum and the pollen grains) from other maternal cell layers. Furthermore ,the authors proved that two NADPH oxidases, RBOHE and RBOHC, are required for the formation and barrier functions of the PTM. The pollen wall forming genes are needed to consolidate the structure of PTM. Overall this is an interesting work with well-designed experiments, providing the insight into how plant male organ anther forms structure for chemical delivery and signaling during pollen formation.

Comment: I am not fully convinced by the statement that the middle layer appears to be key player for the PTM formation by supplying specific phenolic compounds. I assume that tapetum may also has a role in synthesizing PTM as tapetum is a active tissue for the synthesis of phenolic compounds.

***We agree that this has been overstated, particularly for later stages of PTS biogenesis, and have toned down our statement accordingly. However, our expression analysis does suggest that at early stages, the middle layer (and potentially the endothecium), are likely to be the major site of biosynthesis of phenylpropanoid precursors.***

References

1. Truskina, J. *et al.* A peptide-mediated, multilateral molecular dialogue for the coordination of pollen wall formation. *Proc Natl Acad Sci U S A* **119**, e2201446119 (2022).

2. Clément, C. & Audran, J. C. Anther wall layers control pollen sugar nutrition inLilium. *Protoplasma* **187**, 172–181 (1995).

3. Owen, H. A. & Makaroff, C. A. Ultrastructure of microsporogenesis and microgametogenesis inArabidopsis thaliana (L.) Heynh. ecotype Wassilewskija (Brassicaceae). *Protoplasma* **185**, 7–21 (1995).

---

Second decision letter

MS ID#: DEVELOP/2022/200596

MS TITLE: Anther development in Arabidopsis thaliana involves symplastic isolation and apoplastic gating of the tapetum-middle layer interface.

AUTHORS: Jekaterina Truskina, Sophy Boeuf, Joan Renard, Tonni Grube Andersen, Niko Geldner, and Gwyneth Ingram

I have now received all the referees reports on the above manuscript, and have reached a decision. The referees' comments are appended below, or you can access them online: please go to BenchPress and click on the 'Manuscripts with Decisions' queue in the Author Area.

© 2022. Published by The Company of Biologists under the terms of the Creative Commons Attribution License (https://creativecommons.org/licenses/by/4.0/). 

The overall evaluation is positive and we would like to publish a revised manuscript in Development, provided that the referees' comments can be satisfactorily addressed. Please attend to all of the reviewers' comments in your revised manuscript and detail them in your point-by-point response. If you do not agree with any of their criticisms or suggestions explain clearly why this is so. If it would be helpful, you are welcome to contact us to discuss your revision in greater detail. Please send us a point-by-point response indicating your plans for addressing the referee's comments, and we will look over this and provide further guidance.

Reviewer 1

*Advance summary and potential significance to field*

The authors have convincingly characterised that the PTS regulates apoplastic permeability between the tapetum and middle layers, and that its integrity relies on RBOHC and RBOHE. The paper suggests the PTS has complex composition and comprises of lignin-like material and establishes a framework of mutants that might be used to further dissect the composition and function of this barrier in future studies.

*Comments for the author*

The authors have made the manuscript much clearer and better by including the information regarding promoter activity in wild-type and mutant backgrounds.
They have addressed most of my comments. I like the new name, especially given the functional analogy to the casparian strip.

Major Comments:
Regarding quantification - I won't die on the quantification hill, but I think it would aid further exploration of PTS composition with the methods used here moving forward. I understand that heterogeneity can be a hindrance to quantification. However, following the argument that different compounds will have different fluorescence activations and emissions, and therefore that the fluorescence from different wavelengths suggests differences in composition then the relative fluorescence between wavelengths carries important information.
I won't insist on this, but if this were my data, I would draw multiple transects across the images and measure the peaks (PTS associated signal?) and troughs (background) of fluorescence (intensity). I would use the difference as a metric for PTS fluorescence and generate ratios to the same measurements from different wavelengths. Such measurements would quantify differences between Col-0, dex2, ms2 and maybe pksb. However, I acknowledge that this won't capture all the features of the PTS phenotypes and I am naïve to the variation in the images - if my suggestion displays a gross lack of appreciation for the system I apologise!
Vacuolar accumulation of aTP-mTQ2 - The new images in Fig S2 have raised a new consideration. The micrographs that show mTQ2 distribution in the rbohe rbohc mutant, transformed with pGSO2-aTP-mTQ2, look to me to be showing vacuolar accumulation of mTQ2. This is suggested by the silhouette of the nuclei of the fluorescent cells. Apoplastic fluorescence would not trace the nucleus. This prompted me to look harder at the images in Fig 2D, and here too the fluorescent signal is plausibly from accumulation of mTQ2 in the vacuole. Scrutiny of the images in 2C in which the same fusion is expressed from the pAMS promoter suggests there could be fluorescence internal to the cells in addition to the high accumulation in the apoplast. If this is the case, it suggests either that secretion via aTP occurs differently in the different cell types, the different promoters somehow influence targeting of the protein, that the cell wall around the middle layer cells is so different to allow rapid and extensive diffusion (thus diluting the signal), the cell wall of midlle cells is of very low pH (lower than the pKa of mTQ2) or?? Unfortunately, I no longer know what to make of the images from pGSO2-aTP-mTQ2 experiments as I'm not sure the signal detected and shown is apoplastic. If the expression profile is discrete, then could this explain why no aTP-mTQ2 apoplast mobility is observed when expressed from the pGSO2 promoter? Can the authors clarify?
I want to stress that I don't think this undermines the conclusion that the loss of rbohe and c changes PTS permability given the pAMS-aTP-mTQ2 experiments are clear and convincing. However, it does suggest that any conclusions regarding directionality of PTS gating are over-interpretations.

© 2022. Published by The Company of Biologists under the terms of the Creative Commons Attribution License (https://creativecommons.org/licenses/by/4.0/). 

Minor comments:
1. Figure 1 - the labels in panel A are missing. What arrow points to what?
2. Figure 1 - labelling the images with the wavelengths would aid comprehension
3. Figure 2/3A-B - What are the panels? Are they different stages of development?
4. Figure 2 - The PMC label is missing 5. Figure 3 C-D - labelling the images with the wavelengths would aid comprehension 6. Figure 4A-H - labelling the rows with developmental stage would be helpful 7. Figure 5 - labelling the rows with the wavelengths would aid comprehension

Reviewer 2

*Advance summary and potential significance to field*

The revised manuscript provides more strong date supporting the existence and of an apoplastic structure peritapetal membrane between tapetum and surrounding cell layers during pollen development.

*Comments for the author*

The authors addressed all my concerns. I have no more comments.

---

**Second revision**

Author response to reviewers' comments

Responses to reviewers' comments

Reviewer 1

Regarding quantification - I won't die on the quantification hill, but I think it would aid further exploration of PTS composition with the methods used here moving forward. I understand that heterogeneity can be a hindrance to quantification. However, following the argument that disferent compounds will have different fluorescence activations and emissions, and therefore that the fluorescence from different wavelengths suggests differences in composition then the relative fluorescence between wavelengths carries important information.

We totally agree with this reviewer that important information is contained within our images. However, we have discussed at length with experts in the field, and they are unanimous in their opinion that a full comprehension of this information would be very difficult to obtain given the position of the PTS, its extreme thinness, and its heterogeneity. Specific absorption and emission spectra are difficult (impossible) to attribute unequivocally to different phenolic polymers under these conditions. In summary, we could report differences in intensity at different wavelengths, but this would not take us further in understanding their origin.

I won't insist on this, but if this were my data, I would draw multiple transects across the images and measure the peaks (PTS associated signal?) and troughs (background) of fluorescence (intensity). I would use the difference as a metric for PTS fluorescence and generate ratios to the same measurements from different wavelengths. Such measurements would quantify differences between Col-0, dex2, ms2 and maybe pksb. However, I acknowledge that this won't capture all the features of the PTS phenotypes and I am naïve to the variation in the images - if my suggestion displays a gross lack of appreciation for the system I apologise!

Response: We considered carrying out this very procedure. However, as stated previously, the heterogeneity of the signal at cell junctions, in our opinion, makes this type of analysis uninformative. In addition, the changes in distribution between some genotypes are can best be fully appreciated though looking at the images themselves. We feel that quantification would be

© 2022. Published by The Company of Biologists under the terms of the Creative Commons Attribution License (https://creativecommons.org/licenses/by/4.0/).

misleading, especially since the consequences of key factors, such as the degree of polymer crosslinking on emission is not currently known.

Vacuolar accumulation of aTP-mTQ2 - The new images in Fig S2 have raised a new consideration. The micrographs that show mTQ2 distribution in the rbohe rbohc mutant, transformed with pGSO2-aTP-mTQ2, look to me to be showing vacuolar accumulation of mTQ2. This is suggested by the silhouette of the nuclei of the fluorescent cells. Apoplastic fluorescence would not trace the nucleus. This prompted me to look harder at the images in Fig 2D, and here too the fluorescent signal is plausibly from accumulation of mTQ2 in the vacuole. Scrutiny of the images in 2C in which the same fusion is expressed from the pAMS promoter suggests there could be fluorescence internal to the cells in addition to the high accumulation in the apoplast. If this is the case, it suggests either that secretion via aTP occurs differently in the different cell types, the different promoters somehow influence targeting of the protein, that the cell wall around the middle layer cells is so different to allow rapid and extensive diffusion (thus diluting the signal), the cell wall of midlle cells is of very low pH (lower than the pKa of mTQ2) or?? Unfortunately, I no longer know what to make of the images from pGSO2-aTP-mTQ2 experiments as I'm not sure the signal detected and shown is apoplastic. If the expression profile is discrete, then could this explain why no aTP-mTQ2 apoplast mobility is observed when expressed from the pGSO2 promoter? Can the authors clarify?

Response: We thank the reviewer for this comment. We agree that much of the signal in the pGSO2-aTP-mTQ2 lines is cytoplasmic or vacuolar, although some apoplastic signal is visible at the interface between the middle layer and the tapetum. We also see signal within the cells of the tapetum in our pAMS-aTP-mTQ2 lines, but it is clear in these lines most of the signal is apoplastic, possibly because the tapetum is fundamentally a tissue that is "designed" to secrete. Because of these comments we have changed the text.

On Page 6 we have changed our text as follows:
Original text: To test movement in the opposite direction, we expressed the aTP-mTQ2 protein fusion in the middle layer under the GSO2 promoter. In these lines aTP-mTQ2 was detected strongly in and around the cells of the middle layer but also around endothecium and epidermis cells, but no signal was detected in the locular matrix or around the tapetum cells (Fig. 2D). These experiments indicate that the diffusion of apoplastic proteins is restricted at the middle layer - tapetum interface. Combined our data, therefore support the hypothesis that the PTS may prevent diffusion of larger molecules (such as fluorescent proteins) between the ZPD and surrounding sporophytic tissues.

New text: To test movement in the opposite direction, we expressed the aTP-mTQ2 protein fusion in the middle layer under the GSO2 promoter. In these lines aTP-mTQ2 was detected strongly in the cells of the middle layer but also in endothecium and epidermis cells. Some apoplastic signal was also detected around these cells, but no signal was detected in the locular matrix or around the tapetum cells (Fig. 2D). The apparently poor secretion of aTP-mTQ2 by middle layer cells compared to the tapetum, may be a consequence of their different biological functions, and makes interpretation of pGSO2-aTP-mTQ2 lines complex. Nonetheless, our experiments suggest that the diffusion of apoplastic proteins is restricted at the middle layer - tapetum interface, supporting the hypothesis that the PTS may prevent diffusion of larger molecules (such as fluorescent proteins) between the ZPD and surrounding sporophytic tissues.

On Page 8 we have changed the text as follows:
Original text: When aTP-mTQ2 was expressed in the middle layer using pGSO2 in the rbohe rbohc double mutant background the aTP-mTQ2 signal was observed in the middle layer, endothecium and epidermis. The GSO2 promoter was expressed normally in this background (Figure S4). However, no obvious signal was observed in the tapetum or locular matrix (Suppl. Fig. S2B). This apparently contradictory result may be due to the relatively low activity of the GSO2 promoter compared to that of AMS, combined with the technical constraints of observing low quantities of mTQ2 signal within the extensive apoplast of the ZPD.

New text: When aTP-mTQ2 was expressed in the middle layer using pGSO2 in the rbohe rbohc double mutant background, as in wild-type plants the aTP-mTQ2 signal was observed in the middle layer, endothecium and epidermis, predominantly within cells. The GSO2 promoter was expressed normally in this background (Figure S4). However, no obvious signal was observed in the tapetum or

© 2022. Published by The Company of Biologists under the terms of the Creative Commons Attribution License (https://creativecommons.org/licenses/by/4.0/).

locular matrix (Suppl. Fig. S2B). This apparently contradictory result may be due to the relatively low activity of the GSO2 promoter compared to that of AMS, or, as discussed above the apparent lack of strong secretion of aTP-mTQ2 from middle layer cells, combined with the technical constraints of observing low quantities of mTQ2 signal within the extensive apoplast of the ZPD.

I want to stress that I don't think this undermines the conclusion that the loss of rbohe and c changes PTS permability given the pAMS-aTP-mTQ2 experiments are clear and convincing. However, it does suggest that any conclusions regarding directionality of PTS gating are over-interpretations.

Minor comments:
1. Figure 1 - the labels in panel A are missing. What arrow points to what?
2. Figure 1 - labelling the images with the wavelengths would aid comprehension
3. Figure 2/3A-B - What are the panels? Are they different stages of development?
4. Figure 2 - The PMC label is missing
5. Figure 3 C-D - labelling the images with the wavelengths would aid comprehension
6. Figure 4A-H - labelling the rows with developmental stage would be helpful
7. Figure 5 - labelling the rows with the wavelengths would aid comprehension

Response: We thank the reviewer for their comments. We think that these problems must be linked to problems with the PDF conversion/image viewing, as all the requested labels are present in our original images. We are sorry for these issues.

Reviewer 2 Comments for the author

The authors addressed all my concerns. I have no more comments.

---

<u>Third decision letter</u>

MS ID#: DEVELOP/2022/200596

MS TITLE: Anther development in Arabidopsis thaliana involves symplastic isolation and apoplastic gating of the tapetum-middle layer interface.

AUTHORS: Jekaterina Truskina, Sophy Boeuf, Joan Renard, Tonni Grube Andersen, Niko Geldner, and Gwyneth Ingram
ARTICLE TYPE: Research Article

I am happy to tell you that your manuscript has been accepted for publication in Development, pending our standard ethics checks.

<u>Reviewer 1</u>

*Advance summary and potential significance to field*

The text changes acknowledge my concerns regarding the vacuolar accumulation of fluorescence in the tapetum, and these versions of the figures seem to have many labels not previously visible to me.

*Comments for the author*

I have nothing further to add

© 2022. Published by The Company of Biologists under the terms of the Creative Commons Attribution License (https://creativecommons.org/licenses/by/4.0/).
